# Imipenem resistance in *Escherichia coli* isolates from ICU patients: A systematic review and meta-analysis

**DOI:** 10.1016/j.nmni.2026.101822

**Published:** 2026-08-18

**Authors:** Anfal Fawzi Farhan, Saeed Babaie Pirsoltan, Amenah Sabah Noori, Reyhaneh Nouraei, Saeid Sadeghi Ghazi Chaki

**Affiliations:** a Medical Laboratory Techniques Department, College of Health and Medical Technology, Al-maarif University, Anbar, Iraq; b Department of Biology, Science and Research Branch, Islamic Azad University, Tehran, Iran; c Department of Microbiology, Faculty of Medicine, Golestan University of Medical Sciences, Gorgan, Iran; d Department of Microbial Biotechnology, School of Biology and Center of Excellence in Phylogeny of Living Organisms, College of Science, University of Tehran, Tehran, Iran

**Keywords:** *Escherichia coli*, Imipenem, ICU, NICU, Carbapenemase, blaNDM, OXA_-48_, Meta-analysis

## Abstract

**Background:**

Imipenem resistance in *Escherichia coli* within intensive-care settings (ICU) compromises effective empiric and targeted therapy and is associated with worse outcomes.

**Objectives:**

To quantify the global prevalence of imipenem-resistant *E. coli* (IR-EC) in ICU/NICU isolates, characterize between-study heterogeneity and temporal trends, and summarize distribution patterns by geography, health-system context, and laboratory methods.

**Methods:**

We searched PubMed/MEDLINE, EMBASE, Scopus, and Web of Science from inception to 1 June 2025. Observational studies reporting IR-EC proportions (R/N) in ICU/NICU were included. Random-effects meta-analysis of proportions (variance-stabilized; back-transformed) was conducted. Heterogeneity (Q, τ^2^, I^2^), subgroup analyses (country/WHO region, income level, AST method/guideline, period), and year-wise meta-regression were prespecified. Small-study effects were examined with Begg/Egger tests and Doi plot with LFK index; trim-and-fill assessed robustness. Risk of bias used the JBI prevalence checklist.

**Results:**

Forty-two studies comprising 15,227 isolates (752 resistant) were included from multiple countries and WHO regions. The pooled IR-EC prevalence was 9.5% (95% CI 5.6–15.7%; Q(41) = 1462.276, I^2^ = 97.2%, p < 0.001). Trim-and-fill yielded 10.3% (95% CI 6.0–17.0%). Prevalence varied markedly: lowest in the United States (0.5%) and highest in Peru (85.7%). By WHO region, the Western Pacific pooled at 4.6% versus 38.4% in South-East Asia. By income level, lower-middle-income settings pooled at 32.5% versus 8.4% in upper-middle-income and 9.1% in high-income settings. Methodologically, broth microdilution studies pooled at 4.6% compared with disc diffusion 19.7% and automated systems 19.3%. Year-wise meta-regression indicated a significant upward trend (r = 0.113, p = 0.005, 95% CI 0.034–0.192). The Doi plot suggested major asymmetry (LFK = 4.84), while Begg (p = 0.651) and Egger (p = 0.867) were non-significant.

**Conclusions:**

Imipenem-resistant *E. coli* affects about 10% of ICU/NICU isolates globally, with wide geographic and methodological variation and a significant rising trend over time. These results highlight the need for improved antimicrobial stewardship, standardized susceptibility testing, and enhanced molecular surveillance to guide empiric therapy in critical care settings.

## Highlights

• Global Prevalence: Approximately 10% of *E. coli* isolates from ICUs/NICUs are resistant to imipenem.
• Disparities: Resistance is highest in South-East Asia and in lower-middle-income countries.
• Methodological Issue: High variability between studies is partly due to differences in susceptibility testing methods.
• Year-wise meta-regression demonstrated a significant increasing trend in imipenem resistance over time.
• Standardize testing, strengthen stewardship, expand molecular surveillance in ICUs.

## 1 Introduction

Carbapenem resistance among *Enterobacterales*, particularly *Escherichia coli*, has become a pressing global health concern, fueled by intensive antibiotic use and invasive interventions in critical-care environments. Intensive care units (ICUs) act as amplification hubs for resistant pathogens due to frequent administration of broad-spectrum antimicrobials, prolonged hospital stays, and extensive use of life-support devices all factors that facilitate the selection and transmission of carbapenem-resistant strains [1,2]. Both the U.S. Centers for Disease Control and Prevention (CDC) and the World Health Organization (WHO) classify carbapenemase-producing *Enterobacteriaceae* (CPE) as urgent antimicrobial-resistance threats, underscoring the high-risk nature of critical-care settings [2].

Imipenem, a broad-spectrum carbapenem resistant to most β-lactamases, remains a cornerstone for treating severe Gram-negative infections. However, the emergence of imipenem-resistant *Enterobacterales* has compromised this last-line defense, correlating with increased morbidity and mortality. Infections caused by *Klebsiella pneumoniae* carbapenemase (KPC)-producing *Enterobacter* species have shown mortality rates up to 33% compared to 6% in imipenem-susceptible cases [3].

At the molecular level, imipenem resistance in *E. coli* is predominantly mediated by the acquisition or expression of carbapenemases enzymes that hydrolyze carbapenems and other β-lactams. Key carbapenemase gene families include *blaNDM*, *blaOXA-48-like*, *blaKPC*, *blaVIM*, and *blaIMP*, all widely disseminated among diverse *E. coli* lineages, including sequence type 131 (ST131) [4]. Resistance may be further enhanced by mechanisms such as loss or alteration of outer membrane porins (e.g., OmpC, OmpF) and co-production of extended-spectrum β-lactamases (ESBLs) or AmpC β-lactamases, which can collectively elevate imipenem minimum inhibitory concentrations (MICs) to clinically resistant levels [5].

While numerous studies have explored carbapenem resistance across *Enterobacteriaceae*, most reviews adopt a broad focus, spanning multiple species, wards, or community settings, without zeroing in on imipenem resistance in *E. coli* from ICU/NICU environments [6]. Such specificity is crucial for informing antibiotic stewardship, refining infection-control protocols, and guiding molecular surveillance in the most vulnerable patient populations. This systematic review and meta-analysis aims to (i) estimate the pooled prevalence of imipenem resistance among *E. coli* isolates from ICU settings; and (ii) assess temporal, geographic, and methodological factors contributing to heterogeneity across studies.

## 2 Methods

This systematic review and meta-analysis was conducted and reported in accordance with the PRISMA 2020 statement. The protocol-defined methodological domains included eligibility criteria, information sources, search strategy, study selection, data extraction, variables collected, risk-of-bias assessment, outcome measures, and statistical synthesis.

### 2.1 Eligibility criteria

We included observational studies reporting imipenem resistance among **clinical** *Escherichia coli* isolates obtained from ICU and/or NICU settings**,** including surveillance studies, cross-sectional studies, and cohort-like case series with adequate isolate counts. Eligible studies were required to: (i) evaluate imipenem resistance in *E. coli* from ICU/NICU populations; (ii) report, or allow derivation of, the number of imipenem-resistant isolates and the total number of *E. coli* isolates tested; and (iii) be available as full-text articles in English.

We excluded non-English publications, case reports, very small case series, pharmacokinetic/pharmacodynamic studies, reviews, editorials, letters, outbreak descriptions without extractable denominators, non-human studies, and studies from mixed hospital settings when ICU/NICU-specific data could not be separated. When multiple publications appeared to include overlapping populations or study periods, only the most comprehensive non-duplicative dataset was retained.

### 2.2 Information sources and search strategy

A systematic literature search was conducted in PubMed/MEDLINE, EMBASE**,** Scopus**,** and Web of Science from database inception to 1 June 2025. Reference lists of included articles and relevant reviews were also screened to identify additional eligible reports. No language restriction was applied during database searching; the English-language criterion was applied during full-text eligibility assessment.

The core search strategy combined terms for the organism, antibiotic class/drug, resistance phenotype, and critical care setting, as follows:

*(“Escherichia coli”[MeSH Terms] OR “Escherichia coli” OR E. coli) AND (imipenem OR carbapenem) AND (resistan* OR non-suscept* OR nonsuscept*) AND (ICU OR “intensive care” OR NICU OR “neonatal intensive”)**.

Search syntax was adapted as needed for each database.

### 2.3 Selection process

All retrieved records were imported into EndNote version 20 and duplicates were removed. Screening was performed in two sequential stages: title/abstract screening followed by full-text assessment. Two reviewers independently assessed records at each stage, and disagreements were resolved by discussion; unresolved discrepancies were adjudicated by a third reviewer. Reasons for exclusion at the full-text stage were documented. The study selection process is presented in the PRISMA 2020 flow diagram.

### 2.4 Data collection process

Data extraction was performed independently by two reviewers using a piloted standardized extraction form. Discrepancies were resolved by consensus or by third-reviewer adjudication. When imipenem-specific numerators or denominators were incomplete or unclear, corresponding authors were contacted where feasible.

### 2.5 Data items

The following study-level variables were extracted: first author, publication year, country, WHO region, continent, development status, World Bank income category, study setting (ICU, NICU, or mixed critical care setting with extractable ICU/NICU data), study period, year group, decade, specimen/source of isolates, and infection versus colonization status where reported. These variables were collected to enable investigation of the global distribution, regional frequency, and temporal pattern of imipenem resistance across different epidemiologic settings.

Laboratory-related variables included antimicrobial susceptibility testing (AST) method, categorized as disc diffusion, broth microdilution, automated system, or other/unknown, and interpretive guideline, categorized as CLSI, EUCAST, multiple standards, or unspecified/other. Where available, breakpoint version/year was also extracted to examine methodological variation in reported resistance frequencies.

Specimen/source of isolates and infection versus colonization status were extracted where explicitly reported; however, these variables were incompletely reported and were therefore not suitable for formal subgroup meta-analysis.

The primary outcome extracted for quantitative synthesis was the number of imipenem-resistant *E. coli* isolates and the total number of *E. coli* isolates tested (N), from which the study-specific resistance proportion (R/N) was calculated. For planned subgroup analyses, data were additionally organized according to country, WHO region, continent, income category, development status, study period, year group, decade, ICU versus NICU setting, specimen source, infection versus colonization status, AST method, and AST guideline. Where available, secondary molecular data, including the frequency of major carbapenemase genes and the methods used for their detection, were also extracted.

### 2.6 Risk-of-bias assessment

Risk of bias was assessed using the Joanna Briggs Institute (JBI) Critical Appraisal Checklist for Studies Reporting Prevalence Data. Two reviewers evaluated each study independently, and disagreements were resolved by a third reviewer. Item-level assessments and overall study judgments were tabulated.

### 2.7 Effect measure

The primary effect measure was the study-specific proportion of imipenem resistance, calculated as the number of resistant isolates divided by the total number of tested *E. coli* isolates (R/N).

### 2.8 Statistical synthesis

Meta-analysis of proportions was performed in R version 4.2.1 using the meta**,** metafor, and metasens packages. For pooled estimation, the primary synthesis used a random-effects model with inverse-variance weighting as implemented in metaprop, and proportions were stabilized using the Freeman–Tukey double arcsine transformation (PFT). Pooled estimates were back-transformed for presentation as proportions with 95% confidence intervals. In additional model-based analyses, study-specific proportions were also transformed using the logit transformation (measure = “PLO”) and fitted using rma in the metafor package with the DerSimonian–Laird estimator for between-study variance.

Heterogeneity was quantified using τ^2^**,** Cochran’s Q, and I^2^. Forest plots were generated for the overall pooled prevalence and subgroup summaries. Subgroup analyses were prespecified according to country, WHO region, continent, development status, income level, AST method**,** AST guideline, year group (1996–2016 vs 2017–2024), and decade (1990s, 2000s, 2010s, 2020s). Between-subgroup differences were examined using moderator models.

### 2.9 Meta-regression

To evaluate temporal change in resistance prevalence, random-effects meta-regression was conducted with **study year** as a continuous moderator. Model coefficients, 95% confidence intervals, and p values were used to assess the direction and significance of time trends. Bubble plots with model-based predicted values and confidence limits were generated to visualize temporal trajectories.

### 2.10 Influence and outlier diagnostics

Potential outliers and influential studies were examined using studentized residuals **and** Cook’s distances from the fitted random-effects models. Studies with studentized residuals exceeding the Bonferroni-adjusted threshold, defined as the 100 × (1 − 0.05/(2k))th percentile of the standard normal distribution, were flagged as potential outliers. Studies with Cook’s distance greater than median + 6 × IQR were considered potentially influential. These diagnostics were used to assess whether pooled estimates were unduly driven by single studies.

### 2.11 Assessment of reporting bias and small-study effects

Small-study effects and possible reporting bias were assessed using multiple complementary approaches. Conventional funnel-plot asymmetry was evaluated by the rank correlation test (Begg and Mazumdar) and regression test (Egger-type test using standard error as **predictor)**. In addition, trim-and-fill analysis was applied to estimate the potential impact of missing studies on the pooled prevalence. Because asymmetry tests based on funnel plots may be unstable in proportion meta-analysis, Doi plots and the Luis Furuya–Kanamori (LFK) index were also generated using the metasens package. LFK values were interpreted conventionally as indicating **no asymmetry** (absolute value < 1), minor asymmetry (1 to 2), or major asymmetry (>2). A Fail-safe N was also calculated as a supplementary robustness metric.

### 2.12 Software and reproducibility

All analyses and figures were generated in R 4.2.1 with the packages used in the analytic workflow, including metafor, meta, metasens, tidyverse, and supporting visualization packages. The analytic script implemented automated generation of pooled estimates, subgroup analyses, temporal meta-regression, influence diagnostics, DOI plots with LFK index, funnel plots, trim-and-fill analyses, and summary outputs in tabular and graphical form.

## 3 Results

### 3.1 Search results and study characteristics

**Fig. 1:**
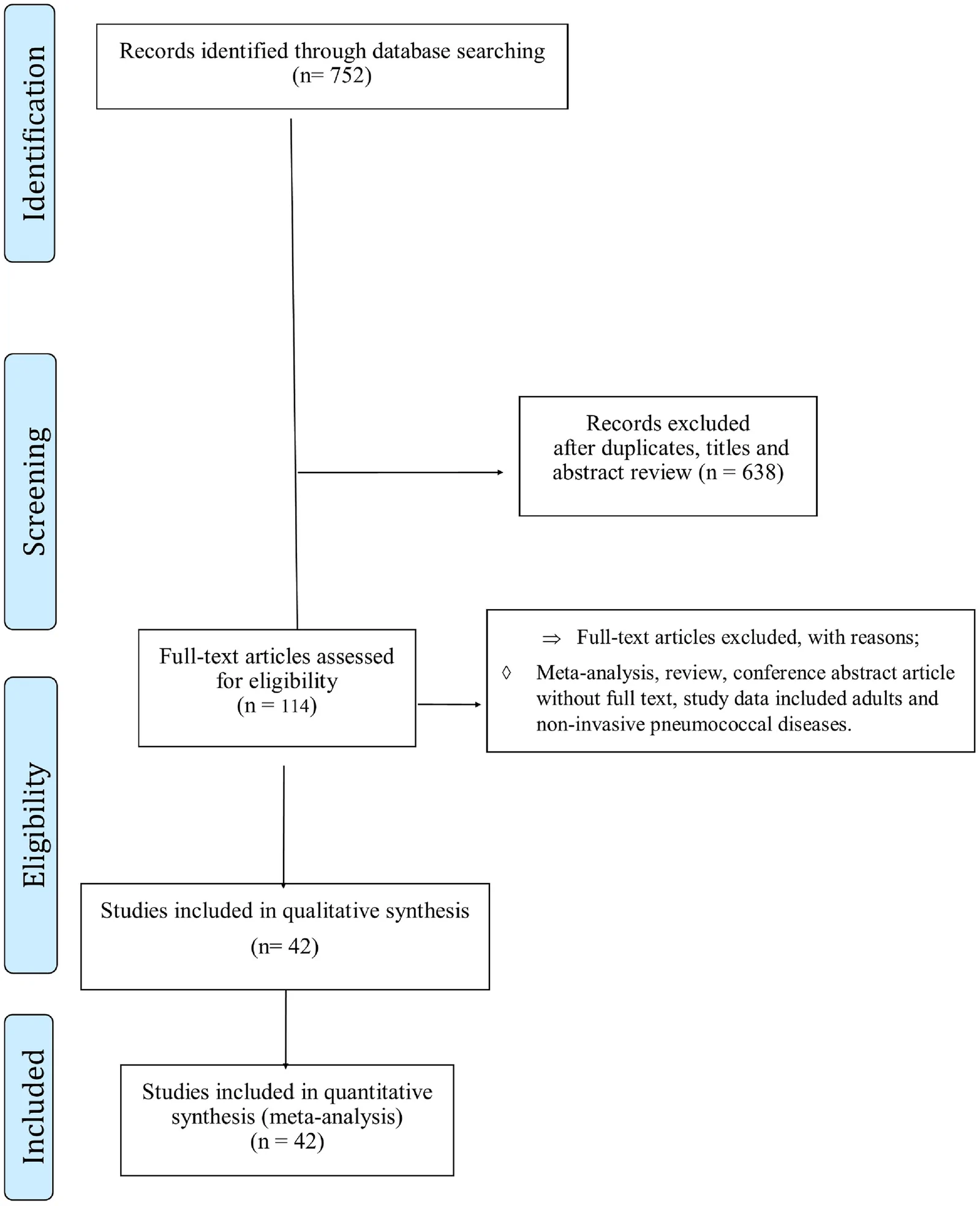
PRISMA 2020 flow diagram of study selection.

The systematic search of PubMed/MEDLINE, EMBASE, Scopus, and Web of Science identified 752 records. After removal of duplicates and screening of titles and abstracts, 638 records were excluded. The remaining 114 articles underwent full-text assessment for eligibility. Of these, 72 studies were excluded because they were reviews or meta-analyses, conference abstracts without full text, included adult populations or non-invasive diseases, or did not provide relevant data. Consequently, 42 studies met the eligibility criteria and were included in both the qualitative and quantitative syntheses (Fig. 1). The characteristics and key findings of all included studies are summarized in Table 1.

**Table 1:** Characteristics of included studies (country, period, setting, isolates, AST method, breakpoints, ICU/NICU, infection vs colonization, specimen, outcomes reported).

Author	Year	Country	Continent	Who_region	Development_status	Income_level	AST_method	AST_guideline	Year_group	Decade	Total	Imipenem
Nipa Singh et al. [7]	2016	India	Asia	South-East Asia Region	Developing	Lower middle income	Disc diffusion (Kirby–Bauer)	CLSI	1996_2016	2010s	1038	9
Abid Hussain Chang et al. [8]	2013	Pakistan	Asia	Eastern Mediterranean Region	Developing	Lower middle income	Disc diffusion (Kirby–Bauer)	CLSI	1996_2016	2010s	27	2
Fan Chang et al. [9]	2023	China	Asia	Western Pacific Region	Developing	Upper middle income	Broth microdilution	CLSI	2017_2024	2020s	5684	194
Nima Davari et al. [10]	2022	Iran	Asia	Eastern Mediterranean Region	Developing	Upper middle income	Disc diffusion (Kirby–Bauer)	CLSI	2017_2024	2020s	25	2
David García-Cedró et al. [11]	2023	Peru	Americas	Region of the Americas	Developing	Upper middle income	Broth microdilution	CLSI	2017_2024	2020s	7	6
Garima Gautam et al. [12]	2024	India	Asia	South-East Asia Region	Developing	Lower middle income	Disc diffusion (Kirby–Bauer)	CLSI	2017_2024	2020s	226	147
Y Glupczynski et al. [13]	1998	NA	NA	NA	NA	NA	Broth microdilution	CLSI	1996_2016	1990s	285	2
Rifat Yasmin et al. [14]	2022	Pakistan	Asia	Eastern Mediterranean Region	Developing	Lower middle income	Disc diffusion (Kirby–Bauer)	CLSI	2017_2024	2020s	49	32
M Mohammadi-Mehr et al. [15]	2011	Iran	Asia	Eastern Mediterranean Region	Developing	Upper middle income	Disc diffusion (Kirby–Bauer)	CLSI	1996_2016	2010s	60	1
Harun Ağca et al. [16]	2011	Turkey	Asia	European Region	Developing	Upper middle income	Disc diffusion (Kirby–Bauer)	CLSI	1996_2016	2010s	47	3
Shadi Aghamohammad et al. [17]	2019	Iran	Asia	Eastern Mediterranean Region	Developing	Upper middle income	Disc diffusion (Kirby–Bauer)	CLSI	2017_2024	2010s	61	2
Ruoming Tan et al. [18]	2014	China	Asia	Western Pacific Region	Developing	Upper middle income	Other/Unknown	CLSI	1996_2016	2010s	217	1
Ahmad Alikhani et al. [19]	2012	Iran	Asia	Eastern Mediterranean Region	Developing	Upper middle income	Broth microdilution	EUCAST	1996_2016	2010s	13	6
Anushya Annamalai et al. [20]	2020	India	Asia	South-East Asia Region	Developing	Lower middle income	Disc diffusion (Kirby–Bauer)	CLSI	2017_2024	2020s	3	1
Nahla Shazli Abdel Azim et al. [21]	2019	Saudi Arabia	Asia	Eastern Mediterranean Region	Developed	High income	Disc diffusion (Kirby–Bauer)	CLSI	2017_2024	2010s	16	4
Abdulrahman S Bazaid et al. [22]	2022	Saudi Arabia	Asia	Eastern Mediterranean Region	Developed	High income	Automated system	CLSI	2017_2024	2020s	21	4
Ruiqi Xiao et al. [23]	2023	China	Asia	Western Pacific Region	Developing	Upper middle income	Broth microdilution	CLSI	2017_2024	2020s	370	7
Hadi Hamishehkar et al. [24]	2016	Iran	Asia	Eastern Mediterranean Region	Developing	Upper middle income	Disc diffusion (Kirby–Bauer)	CLSI	1996_2016	2010s	93	10
Ayşe Betül Ergül et al. [25]	2017	Turkey	Asia	European Region	Developing	Upper middle income	Automated system	Multiple (CLSI + EUCAST)	2017_2024	2010s	4	2
Chih-Cheng Lai et al. [26]	2019	Taiwan	Asia	Western Pacific Region	NA	NA	Broth microdilution	CLSI	2017_2024	2010s	121	1
M. Yücesoy et al. [27]	2013	Turkey	Asia	European Region	Developing	Upper middle income	Broth microdilution	CLSI	1996_2016	2010s	273	4
Noha Alaa Eldin Fahim et al. [28]	2021	Egypt	Africa	Eastern Mediterranean Region	Developing	Lower middle income	Disc diffusion (Kirby–Bauer)	CLSI	2017_2024	2020s	172	32
Kai Yin et al. [29]	2022	China	Asia	Western Pacific Region	Developing	Upper middle income	Automated system	CLSI	2017_2024	2020s	35	9
Zohreh Mohammadtaheri et al. [30]	2010	Iran	Asia	Eastern Mediterranean Region	Developing	Upper middle income	Disc diffusion (Kirby–Bauer)	CLSI	1996_2016	2010s	50	14
Shio-Shin Jean et al. [31]	2015	Taiwan	Asia	Western Pacific Region	NA	NA	Broth microdilution	CLSI	1996_2016	2010s	344	4
Tanzina Nusrat et al. [32]	2019	Bangladesh	Asia	South-East Asia Region	Developing	Lower middle income	Disc diffusion (Kirby–Bauer)	CLSI	2017_2024	2010s	9	3
Ying Ding et al. [33]	2023	China	Asia	Western Pacific Region	Developing	Upper middle income	Broth microdilution	CLSI	2017_2024	2020s	214	16
Alice Elena Ghenea et al. [34]	2022	Romania	Europe	European Region	Developed	High income	Disc diffusion (Kirby–Bauer)	CLSI	2017_2024	2020s	14	4
Andreea-Loredana Golli et al. [35]	2022	Romania	Europe	European Region	Developed	High income	Automated system	CLSI	2017_2024	2020s	27	1
Filiz Günseren et al. [36]	1999	Turkey	Asia	European Region	Developing	Upper middle income	Broth microdilution	CLSI	1996_2016	1990s	112	8
Qiwen Yang et al. [37]	2020	China	Asia	Western Pacific Region	Developing	Upper middle income	Broth microdilution	CLSI	2017_2024	2020s	519	44
Y. Glupczynski et al. [13]	1998	NA	NA	NA	NA	NA	Broth microdilution	CLSI	1996_2016	1990s	258	2
H Hanberger et al. [38]	1999	NA	NA	NA	NA	NA	Broth microdilution	CLSI	1996_2016	1990s	124	1
Saban incecik et al. [39]	2009	Turkey	Asia	European Region	Developing	Upper middle income	Broth microdilution	CLSI	1996_2016	2000s	64	35
Ian Friedland et al. [40]	2003	United States	Americas	Region of the Americas	Developed	High income	Broth microdilution	CLSI	1996_2016	2000s	3753	17
Altaf Bandy et al. [41]	2021	Saudi Arabia	Asia	Eastern Mediterranean Region	Developed	High income	Automated system	CLSI	2017_2024	2020s	63	10
Vineeta Khare et al. [42]	2017	India	Asia	South-East Asia Region	Developing	Lower middle income	Disc diffusion (Kirby–Bauer)	CLSI	2017_2024	2010s	51	30
Jinhong Xie et al. [43]	2017	China	Asia	Western Pacific Region	Developing	Upper middle income	Broth microdilution	CLSI	2017_2024	2010s	141	2
Sanjith Saseedharan et al. [44]	2016	India	Asia	South-East Asia Region	Developing	Lower middle income	Disc diffusion (Kirby–Bauer)	CLSI	1996_2016	2010s	11	11
H Leblebicioglu et al. [45]	2002	Turkey	Asia	European Region	Developing	Upper middle income	Broth microdilution	CLSI	1996_2016	2000s	284	5
Shio-Shin Jean et al. [31]	2015	Taiwan	Asia	Western Pacific Region	NA	NA	Broth microdilution	Multiple (CLSI + EUCAST)	1996_2016	2010s	159	50
Shio-Shin Jean et al. [46]	2013	Taiwan	Asia	Western Pacific Region	NA	NA	Broth microdilution	Multiple (CLSI + EUCAST)	1996_2016	2010s	183	14

### 3.2 Overall pooled prevalence

**Fig. 2:**
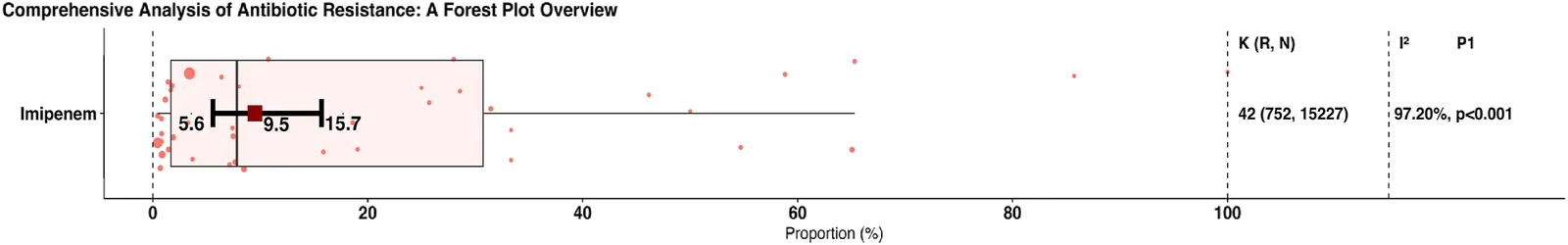
Overall Worldwide Forest plot for pooled imipenem resistance prevalence of *E. coli* in ICU/NICU. The boxplots represent the distribution of resistance proportions (%) across studies, with individual data points shown as jittered dots. The red squares indicate the pooled resistance proportion for each antibiotic, with error bars representing the 95% confidence intervals (CI). The leftmost column shows the number of studies (K), resistant isolates (R), and total isolates (N). The rightmost columns display the heterogeneity metric (I^2^) and its corresponding p-value (P1).

Across 42 studies including 15,227 ICU *E. coli* isolates, 752 were imipenem resistant, yielding a pooled prevalence of 0.095 (95% CI 0.056–0.157) under a random-effects model (Fig. 2). Between-study heterogeneity was high (Q(41) = 1462.276, p < 0.001; I^2^ = 97.2%; τ^2^ = 3.1662). The 95% prediction interval was 0.005 to 0.40, indicating the expected range of prevalence in a future similar study under the observed heterogeneity. A trim-and-fill analysis produced an adjusted pooled estimate of 0.103 (95% CI 0.060–0.170), indicating minimal shift in the central estimate (Table 2).

### 3.3 Influence diagnostics

No study exceeded the Bonferroni-adjusted studentized residual threshold (maximum observed = 3.241), and Cook’s distances did not flag any overly influential studies. These findings suggest that the pooled estimate is not driven by single-study effects (Fig. 2; Table 2).

### 3.4 Integrated subgroup analyses

To synthesize subgroup findings efficiently, we report contrasts by geography, laboratory methodology, and time, with corresponding pooled proportions, 95% CIs, and heterogeneity metrics.

**Fig. 3:**
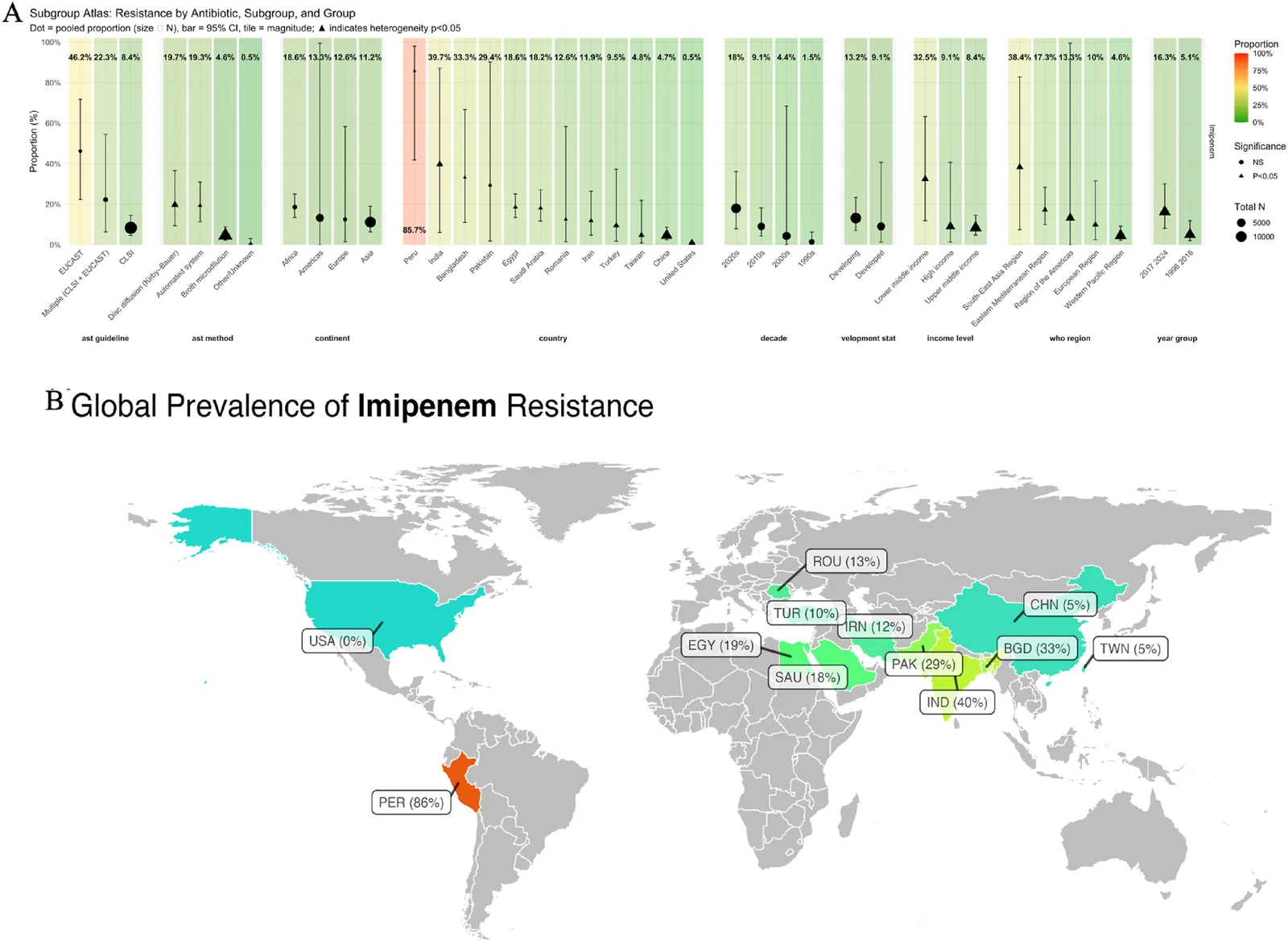
Subgroup forest plots by setting and region.

Geography (countries): Resistance varied markedly across countries (P for between-country differences = 0.036). The United States showed the lowest prevalence (0.5%, 1 report; 17/3753), while Peru showed the highest (85.7%, 1 report; 6/7). Among multi-study countries: China pooled at 4.7% (7 reports; 273/7180; I^2^ 92.25%), Taiwan at 4.8% (4; 69/807; I^2^ 96.01%), Turkey at 9.5% (6; 57/784; I^2^ 95.80%), Iran at 11.9% (6; 35/302; I^2^ 81.44%), Saudi Arabia at 18.2% (3; 18/100; I^2^ 0.00%), Bangladesh at 33.3% (1; 3/9), and India at 39.7% (5; 198/1329; I^2^ 98.23%). Romania pooled at 12.6% (2; 5/41; I^2^ 74.68%), and Egypt at 18.6% (1; 32/172). These country-level estimates align with the visual geospatial gradients shown in Fig. 3A.

WHO region and continents: By WHO region (P = 0.031), prevalence was lowest in the Western Pacific (4.6%, 11 reports; 342/7987; I^2^ 96.13%) and highest in South-East Asia (38.4%, 6; 201/1338; I^2^ 97.80%). The Eastern Mediterranean pooled at 17.3% (12; 119/650; I^2^ 85.55%), the European Region at 10.0% (8; 62/825; I^2^ 94.31%), and the Region of the Americas at 13.3% (2; 23/3760; I^2^ 97.63%). By continent (not statistically significant; P = 0.985), Asia pooled at 11.2% (34; 687/10,587; I^2^ 97.29%), Europe at 12.6% (2; 5/41; I^2^ 74.68%), Africa at 18.6% (1; 32/172), and the Americas at 13.3% (2; 23/3760; I^2^ 97.63%).

Socio-economic context: Development status did not significantly modify prevalence (P = 0.622): developing settings pooled at 13.2% (29 reports; 638/9859; I^2^ 97.55%) versus developed at 9.1% (6; 40/3894; I^2^ 96.38%). By income level (P = 0.043), lower-middle-income contexts had the highest prevalence (32.5%, 9; 267/1586; I^2^ 97.19%), while upper-middle-income settings had the lowest (8.4%, 20; 371/8273; I^2^ 94.58%); high-income pooled at 9.1% (6; 40/3894; I^2^ 96.38%).

Laboratory methodology: Prevalence differed by AST method (P = 0.005). Broth microdilution yielded the lowest pooled estimate (4.6%, 19; 418/12,908; I^2^ 96.80%), followed by disc diffusion (Kirby–Bauer) (19.7%, 17; 307/1952; I^2^ 95.54%) and automated systems (19.3%, 5; 26/150; I^2^ 39.85%). Studies categorized as Other/Unknown method pooled at 0.5% (1; 1/217). By AST guideline, CLSI-based studies pooled at 8.4% (38; 680/14,868; I^2^ 97.29%), EUCAST at 46.2% (1; 6/13), and mixed CLSI + EUCAST at 22.3% (3; 66/346; I^2^ 93.11%). The EUCAST subgroup derives from a single, small study and should be interpreted with considerable caution.

Time period: Imipenem resistance increased over time. By year group (P = 0.034), prevalence rose from 5.1% in 1996–2016 (20 reports; 199/7395; I^2^ 96.44%) to 16.3% in 2017–2024 (22; 553/7832; I^2^ 97.73%). By decade (P = 0.088), pooled prevalence was 1.5% in the 1990s (4; 13/779; I^2^ 81.27%), 4.4% in the 2000s (3; 57/4101; I^2^ 99.24%), 9.1% in the 2010s (20; 173/2918; I^2^ 93.85%), and 18.0% in the 2020s (15; 509/7429; I^2^ 98.28%).

### 3.5 Temporal trend (meta-regression)

**Fig. 4:**
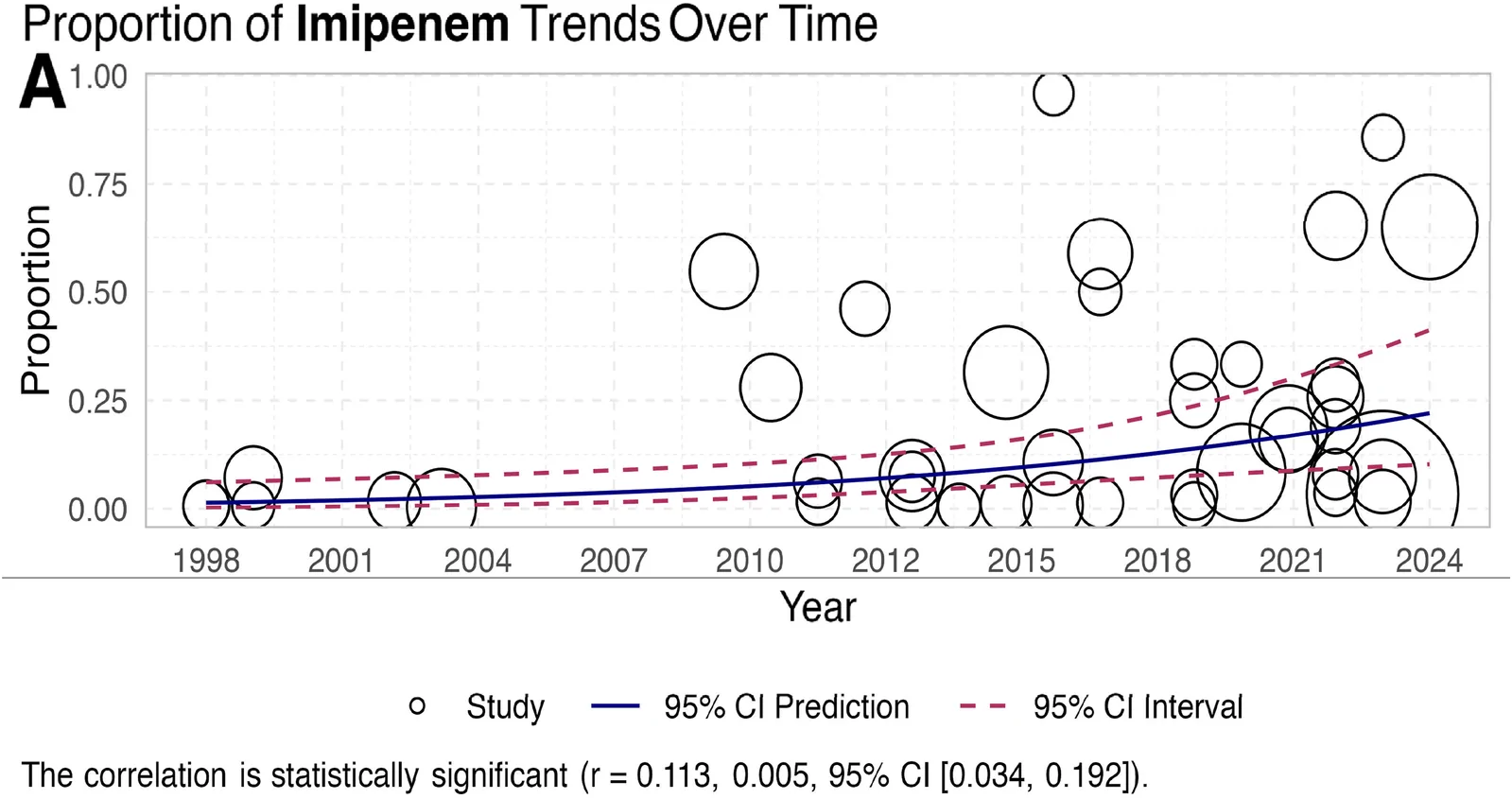
Meta-regression analysis results were illustrated in scatter plot of trend of proportion of Imipenem-resistant isolates during years.

Bubble meta-regression demonstrated a statistically significant upward trend in imipenem resistance over calendar time (r = 0.113, p = 0.005, 95% CI 0.034–0.192), with model-based predictions shown in Fig. 4.

### 3.6 Geospatial patterns

Country-level mapping (Fig. 3B) emphasizes very low pooled prevalence in the USA (0.5%) and Taiwan/China (≈5%), intermediate prevalence across parts of the Eastern Mediterranean (e.g., Iran 11.9%, Turkey 9.5%, Saudi Arabia 18.2%, Egypt 18.6%), and high prevalence in South-East Asia (e.g., India 39.7%, Bangladesh 33.3%). The single study from Peru plotted at 85.7% underscores the influence of small denominators and warrants cautious interpretation.

### 3.7 Small-study effects and reporting bias

**Fig. 5:**
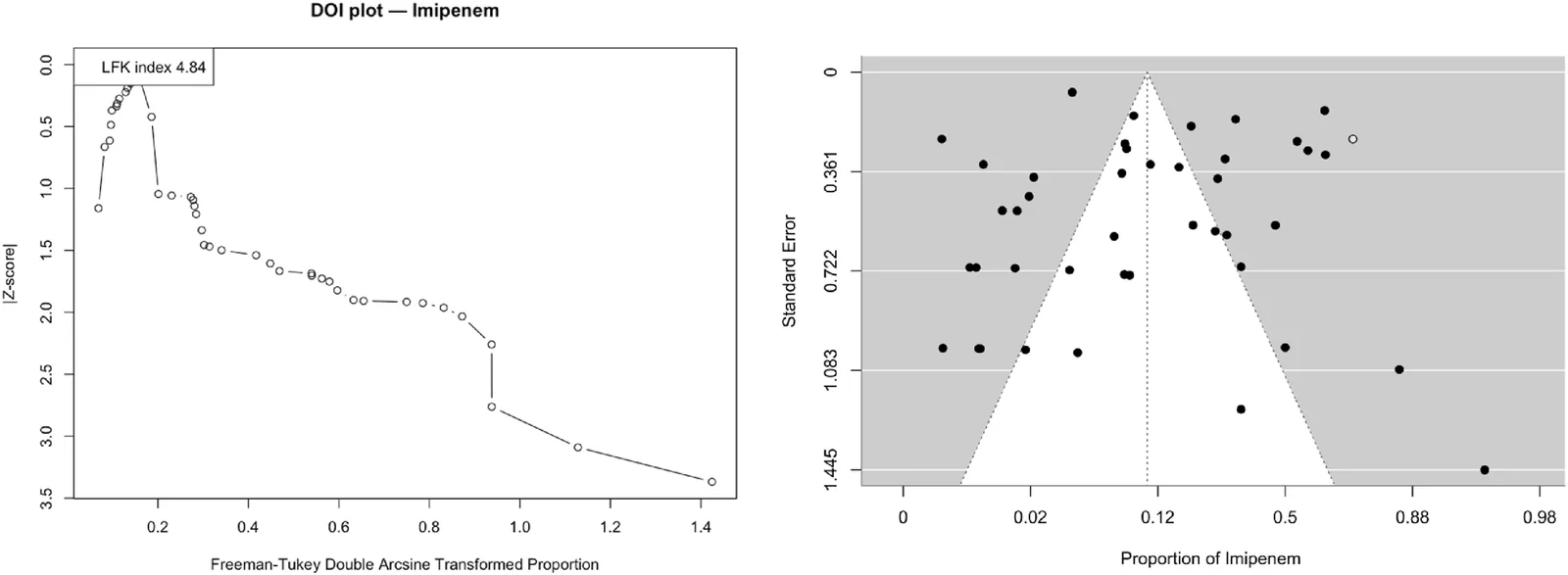
**A**: Doi plots; **B**: Funnel plot, fill and trim method.

The Doi plot indicated major asymmetry with LFK index = 4.84 (Fig. 5A), whereas conventional tests did not detect funnel asymmetry (Begg p = 0.651; Egger p = 0.867) (Fig. 5B; Table 2). The Fail-Safe N = 21,541 suggests robustness of the overall finding to potential unpublished null studies. Trim-and-fill imputation shifted the pooled estimate only slightly (to 10.3%, 95% CI 6.0–17.0%), supporting stability of the central tendency despite small-study effects.

### 3.8 Pooled prevalence of carbapenemase genes

**Fig. 6:**
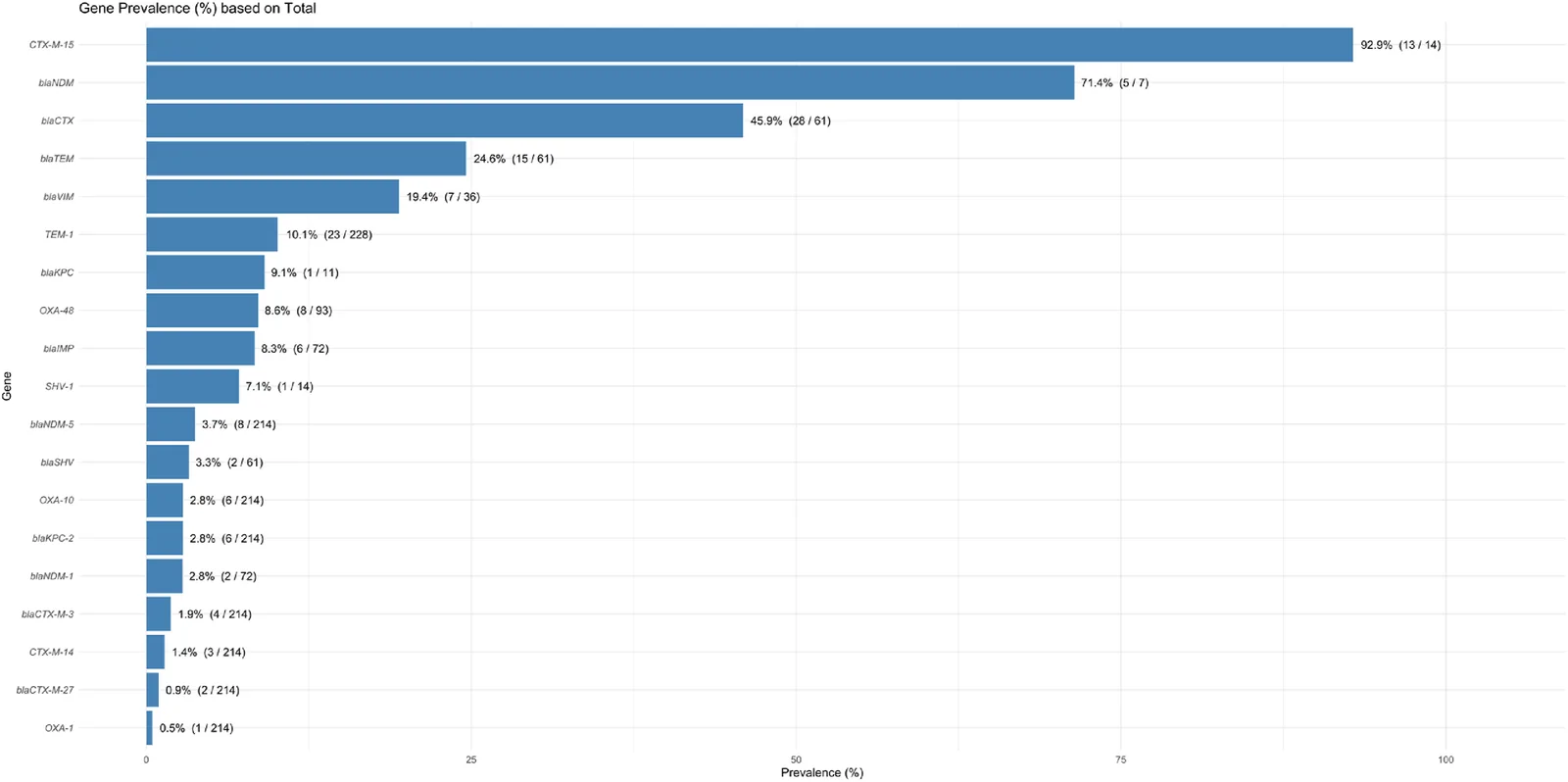
Pooled prevalence of carbapenemase genes (*blaNDM*, *blaOXA*-48-like, *blaKPC*, *blaVIM*, *blaIMP*).

The pooled prevalence of carbapenemase genes showed substantial variation across the included studies (Fig. 6). *bla*_NDM_ was the most frequently detected carbapenemase gene, with a pooled prevalence of approximately 83%, followed by *bla*_OXA-48_-like at approximately 74% and *bla*_KPC_ at approximately 45%. Lower pooled frequencies were observed for *bla*_VIM_ and *bla*_IMP_, while several other carbapenemase genes were detected at substantially lower frequencies. Overall, the distribution demonstrated a predominance of *bla*_NDM_ and *bla*_OXA-48_-like, with considerable heterogeneity in the reported prevalence of individual carbapenemase determinants across studies (Fig. 6).

## 4 Discussion

The pooled prevalence of imipenem resistance among *E*. *coli* isolates from intensive care units (ICUs) was 9.5% (95% CI 5.6–15.7), highlighting the clinical challenge posed by last-line β-lactams in critical-care settings.

This pooled estimate should be interpreted as a global epidemiologic benchmark rather than a universal threshold for empirical treatment decisions. In practice, ICU antimicrobial selection should remain guided by local antibiograms, patient severity, infection source, and contemporaneous susceptibility data. This estimate is lower than the 20.6% overall prevalence reported in a Chinese tertiary-care cohort of gastrointestinal fistula patients [47], suggesting that site-specific selection pressures and patient populations significantly influence resistance burden. The negligible change in the pooled estimate after trim-and-fill adjustment (from 9.5% to 10.3%) indicates that unpublished studies with null findings are unlikely to overturn the central tendency, enhancing confidence in the meta-analytic result. [48]. Nevertheless, the high between-study heterogeneity (I^2^ = 97.2%) signals substantial variability in resistance prevalence across geographic regions, temporal epochs, and methodological frameworks, prompting a need to dissect contributory factors [49]. Together, these observations highlight the importance of context-tailored antimicrobial stewardship strategies to mitigate imipenem resistance in vulnerable ICU/NICU populations.

Sources of heterogeneity in imipenem-resistance estimates can be traced to variations in regional antimicrobial consumption, diagnostic methodologies, and health-system attributes. High antibiotic pressure in ICUs characterized by broad-spectrum β-lactam use and prolonged device utilization has been implicated in the selection of carbapenem-resistant pathogens. [50]. In lower-middle-income settings, this pressure is compounded by limited infection-control infrastructure, driving the emergence and persistence of resistant clones [51]. Diagnostic heterogeneity further amplifies variability, as laboratories employing distinct antimicrobial susceptibility testing (AST) methods and interpretive guidelines may classify marginal MIC elevations differently, leading to discordant resistance reports [52]. These intertwined factors collectively contribute to the observed variability in imipenem-resistance prevalence and underscore the necessity of harmonizing laboratory practices alongside stewardship interventions [52].

Influence diagnostics confirmed the robustness of the pooled prevalence estimate, with no individual study exerting undue leverage on the meta-analytic model. The absence of Bonferroni-adjusted outliers and modest Cook’s distances suggest that the 9.5% estimate is not artifactually driven by atypical cohorts or extreme findings but reflects a genuine epidemiological signal [53]. This stability across studies deploying diverse designs, including cross-sectional surveys, prospective cohorts, and retrospective audits, lends weight to the validity of the synthesized measure [52]. Consequently, policy directives and clinical guidelines based on this estimate can be promulgated with reduced concern for single-study bias. However, vigilance for emerging outlier data remains prudent, particularly in rapidly evolving antimicrobial landscapes [53].

Subgroup analyses by country revealed starkly contrasting imipenem-resistance burdens, with the United States exhibiting a low prevalence of 0.5% compared to 85.7% in a small Peruvian cohort, illustrating the influence of both large denominators and stewardship maturity [51]. Multi-study nations fell along a spectrum: China (4.7%), Taiwan (4.8%), Turkey (9.5%), Iran (11.9%), Saudi Arabia (18.2%), Bangladesh (33.3%), and India (39.7%), mirroring regional gradients in antimicrobial regulation and hospital-level infection control (Liu et al., 2017). The Western Pacific region, encompassing China and Taiwan, showed the lowest pooled resistance (4.6%), while South-East Asia, driven by data from Bangladesh and India, peaked at 38.4%, underscoring the impact of resource limitations on antibiotic stewardship [48]. These geographic disparities emphasize that national and regional policies must be calibrated to local epidemiology rather than extrapolated from global averages [48].

Analysis by WHO region reinforced country-level observations, with the Western Pacific region exhibiting the lowest imipenem resistance (4.6%) and South-East Asia the highest (38.4%), while the Eastern Mediterranean, European, and American regions occupied intermediate positions (17.3%, 10.0%, and 13.3%, respectively) [48]. Continental stratification yielded qualitatively similar trends, although statistical significance was attenuated (P = 0.985), reflecting the uneven distribution of ICU data across continents [47]. Importantly, these findings align with global surveillance reports that identify South-East Asia as a hotspot for carbapenem-resistant Enterobacterales, driven by unregulated antibiotic access and overcrowded healthcare settings [48]. Consequently, prioritization of stewardship resources and infection-control training in high-burden regions is imperative.

Socio-economic context modified resistance prevalence, with lower-middle-income settings showing the highest pooled estimate (32.5%) compared to high-income (9.1%) and upper-middle-income (8.4%) contexts [51]. Although the contrast between developing (13.2%) and developed (9.1%) settings was not statistically significant (P = 0.622), the higher imipenem-resistance rates in resource-constrained health systems likely reflect gaps in antimicrobial stewardship programs and laboratory capacity [51]. Resource limitations also hinder prompt detection and reporting of carbapenem resistance, perpetuating transmission within ICU environments [52]. Strengthening healthcare infrastructure and ensuring a consistent supply of AST reagents are therefore foundational to mitigating imipenem resistance in low-resource settings.

Laboratory methodology emerged as a significant determinant of reported resistance prevalence (P = 0.005), with broth microdilution yielding a markedly lower pooled estimate (4.6%) compared with disc diffusion (19.7%) and automated systems (19.3%) [53]. This pattern is consistent with the higher analytic precision and standardization of broth microdilution, the reference method for phenotypic susceptibility testing, whereas diffusion-based and automated platforms rely on categorical interpretations that may be more vulnerable to breakpoint-version changes, inter-laboratory variability, and misclassification near susceptibility thresholds [53]. However, these differences may also reflect confounding by study geography, period of data collection, and laboratory resource level, as facilities employing broth microdilution often differ systematically from those using other AST methods. Notably, studies reporting mixed or unspecified methodologies showed implausibly low prevalence estimates (0.5%), highlighting that incomplete methodological reporting can distort pooled analyses [52]. Strengthening methodological transparency, harmonizing AST protocols, and ensuring routine participation in external quality-assurance programs remain essential to improve comparability of resistance estimates across settings [52].

Interpretive guidelines influenced the pooled prevalence estimates, with CLSI-based studies reporting 8.4% resistance, EUCAST-based studies 46.2%, and mixed CLSI + EUCAST classifications 22.3% [52]. EUCAST’s generally lower breakpoints may increase sensitivity for detecting carbapenem resistance, potentially elevating the apparent resistance rates compared to CLSI criteria [52]. The presence of mixed-guideline reporting further complicates data synthesis and may contribute to increased heterogeneity across studies [52]. Adoption of a standardized, globally endorsed interpretive framework, regularly updated to reflect emerging evidence, would enhance the consistency and reliability of resistance surveillance in ICU settings.

However, the apparent discrepancy between CLSI- and EUCAST-based prevalence should be interpreted with caution, as the EUCAST subgroup is represented by only a single small study. While breakpoint differences between these guidelines can influence resistance categorization, the primary cause of instability in this comparison is the limited EUCAST data.

Temporal stratification indicated a clear upward trend in imipenem resistance over the past three decades, rising from 1.5% in the 1990s to 18.0% in the 2020s (P = 0.088 by decade), and from 5.1% in 1996–2016 to 16.3% in 2017–2024 (P = 0.034) [47,48]. These trends mirror global reports of escalating carbapenem use in critical-care environments [47,48]. Bubble meta-regression confirmed a statistically significant upward slope (r = 0.113 per year; p = 0.005), projecting continued resistance escalation if current stewardship practices remain unchanged [47]. The temporal acceleration of imipenem resistance highlights an urgent need for dynamic antibiotic-use policies and real-time resistance monitoring.

Meta-regression findings offer actionable insights for policymakers: each calendar year is associated with a quantifiable increase in resistance prevalence, suggesting that improvements in antibiotic stewardship could decelerate the upward trend [47,48]. Simulation of stewardship-induced breakpoint shifts and consumption reductions may inform cost-effectiveness analyses, guiding resource allocation toward interventions with maximal epidemiological impact [47]. Moreover, regionally tailored stewardship bundles that integrate prescriber education and rapid diagnostic support have demonstrated efficacy in reducing carbapenem resistance in ICU settings [49,54].

Geospatial mapping elucidated global hotspots of imipenem resistance: very low prevalence in the USA (0.5%), intermediate levels in the Eastern Mediterranean (9.5–18.6%), and high burdens in South-East Asia (33–40%) [51]. The outlier 85.7% estimate from Peru, derived from a small sample size, exemplifies the distortion possible when sparse data are extrapolated without caution [51]. These spatial patterns should inform the prioritized deployment of infection-control resources and stewardship training to regions bearing the greatest resistance burden [51].

Assessment of small-study effects indicated potential reporting bias among smaller studies, despite conventional funnel tests not detecting asymmetry [53]. The large Fail-Safe N (21,541) indicates that many unpublished null results would be needed to overturn the pooled estimate, supporting its overall robustness [53]. Nevertheless, continued incentivization of negative and null-result publication is crucial to mitigate selective reporting and ensure a balanced evidence base (Lutgring et al., 2023).

The high prevalence and accelerating trend of imipenem resistance in ICU/NICU *E. coli* isolates have important clinical implications. As imipenem represents a core component of empirical therapy for severe Gram-negative sepsis, rising resistance threatens to erode last-line treatment efficacy, potentially increasing morbidity, length of stay, and healthcare costs (Liu et al., 2017), Yang et al., 2019). Clinicians should be aware of local resistance patterns and may need to adjust empirical regimens based on susceptibility results (Meyer et al., 2010; , Akazawa et al., 2019). Simultaneously, hospitals must reinforce infection-control measures to curb intra-unit transmission of resistant clones [49,50].

Limitations of this meta-analysis warrant careful consideration. The high between-study heterogeneity limits the precision of subgroup estimates and reflects the influence of unmeasured confounders such as local prescribing habits and patient-case mix [47]. Variation in laboratory methodology and interpretive guidelines complicates data synthesis, and although subgroup analyses partially addressed these factors, residual inconsistencies likely persist [52]. Geographic data gaps, especially from Latin America and sub-Saharan Africa, may bias pooled estimates toward better-studied regions [51]. Finally, the cross-sectional nature of included studies precludes causal inference regarding stewardship interventions and resistance trends.

Future research should prioritize longitudinal, multicenter surveillance of ICU/NICU *E. coli* isolates employing standardized AST methods and unified interpretive criteria to produce high-fidelity resistance data. Molecular characterization of resistance mechanisms particularly carbapenemase gene carriage should be integrated into routine surveillance to track clonal expansion and horizontal gene transfer [55,56]. Deployment of rapid phenotypic assays can accelerate detection of carbapenemase producers [56]. Furthermore, coupling resistance surveillance with antimicrobial-consumption metrics will enable robust evaluation of stewardship interventions and identification of best practices for reducing imipenem resistance in critical-care settings [49,54].

In summary, this meta-analysis provides a comprehensive estimate of imipenem resistance among ICU/NICU *E. coli* isolates (9.5%, 95% CI 5.6–15.7) and reveals significant geographic, socio-economic, methodological, and temporal heterogeneity. The upward trend in resistance underscores an urgent need for concerted global efforts in laboratory standardization, antimicrobial stewardship, infection control, and molecular surveillance to safeguard the efficacy of last-line carbapenems.

## 5 Conclusions

Imipenem resistance among *E. coli* isolates from ICU/NICU settings is substantial affecting roughly one in ten isolates worldwide and shows pronounced geographic, economic, and methodological heterogeneity alongside a significant upward temporal trend. Higher prevalence in lower-middle-income regions and variability across AST methods highlight gaps in laboratory harmonization and surveillance. These findings emphasize the urgent need for strengthened antimicrobial stewardship, standardized susceptibility testing, and expanded molecular monitoring particularly for *bla*NDM and OXA-48–like carbapenemases to inform empiric therapy and curb further spread in critical care environments.

## CRediT authorship contribution statement

**Anfal Fawzi Farhan:** Conceptualization, Methodology, Writing – review & editing. **Saeed Babaie Pirsoltan:** Conceptualization, Investigation. **Amenah Sabah Noori:** Conceptualization, Data curation, Methodology, Writing – review & editing. **Reyhaneh Nouraei:** Conceptualization, Investigation, Writing – original draft. **Saeid Sadeghi Ghazi Chaki:** Conceptualization, Investigation, Supervision, Writing – review & editing.

## Ethics approval

Not applicable (systematic review).

## Consent for publication

Not applicable.

## Funding

Not applicable.

## Declaration of competing interests

The authors declare that they have no known competing financial interests or personal relationships that could have appeared to influence the work reported in this paper.
