# Supplementary material for: Imipenem resistance in Escherichia coli isolates from ICU patients: A systematic review and meta-analysis
Source: New Microbes New Infect. 2026 Aug 18;73:101822. doi: 10.1016/j.nmni.2026.101822 (PMC13543076; doi:10.1016/j.nmni.2026.101822)
Supplement: Supplementary file 1 [file mmc1.docx]

**Imipenem Resistance in *Escherichia coli* Isolates from ICU Patients: A Systematic Review and Meta-Analysis**

## Abstract

**Background:** Imipenem resistance in Escherichia coli within intensive‑care settings (ICU) compromises effective empiric and targeted therapy and is associated with worse outcomes.

**Objectives:** To quantify the global prevalence of imipenem‑resistant E. coli (IR‑EC) in ICU/NICU isolates, characterize between‑study heterogeneity and temporal trends, and summarize distribution patterns by geography, health‑system context, and laboratory methods.

**Methods:** We searched PubMed/MEDLINE, EMBASE, Scopus, and Web of Science from inception to **1 June 2025**. Observational studies reporting IR‑EC proportions (R/N) in ICU/NICU were included. Random‑effects meta‑analysis of proportions (variance‑stabilized; back‑transformed) was conducted. Heterogeneity (Q, τ², I²), subgroup analyses (country/WHO region, income level, AST method/guideline, period), and year‑wise meta‑regression were prespecified. Small‑study effects were examined with Begg/Egger tests and Doi plot with LFK index; trim‑and‑fill assessed robustness. Risk of bias used the JBI prevalence checklist.

**Results:** Forty‑two studies comprising 15,227 isolates (752 resistant) were included from multiple countries and WHO regions. The pooled IR‑EC prevalence was **9.5%** (95% CI **5.6–15.7%**; **Q**(41)=**1462.276**, **I²**=**97.2%**, **p**<0.001). Trim‑and‑fill yielded **10.3%** (95% CI **6.0–17.0%**). Prevalence varied markedly: lowest in the **United States** (**0.5%**) and highest in **Peru** (**85.7%**). By WHO region, the **Western Pacific** pooled at **4.6%** versus **38.4%** in **South‑East Asia**. By income level, **lower‑middle‑income** settings pooled at **32.5%** versus **8.4%** in **upper‑middle‑income** and **9.1%** in **high‑income** settings. Methodologically, **broth microdilution** studies pooled at **4.6%** compared with **disc diffusion** **19.7%** and **automated systems** **19.3%**. Year‑wise meta‑regression indicated a significant upward trend (r=**0.113**, **p**=**0.005**, 95% CI **0.034–0.192**). The Doi plot suggested major asymmetry (LFK=**4.84**), while Begg (**p**=**0.651**) and Egger (**p**=**0.867**) were non‑significant.

**Conclusions:** Imipenem-resistant *E. coli* affects about 10% of ICU/NICU isolates globally, with wide geographic and methodological variation and a significant rising trend over time. These results highlight the need for improved antimicrobial stewardship, standardized susceptibility testing, and enhanced molecular surveillance to guide empiric therapy in critical care settings.

**Keywords:** Escherichia coli; imipenem; ICU; NICU; carbapenemase; **blaNDM**; OXA_‑48_; meta‑analysis

# Introduction

Carbapenem resistance among Enterobacterales, particularly Escherichia coli, has become a pressing global health concern, fueled by intensive antibiotic use and invasive interventions in critical‑care environments. Intensive care units (ICUs) act as amplification hubs for resistant pathogens due to frequent administration of broad‑spectrum antimicrobials, prolonged hospital stays, and extensive use of life‑support devices all factors that facilitate the selection and transmission of carbapenem‑resistant strains (1, 2). Both the U.S. Centers for Disease Control and Prevention (CDC) and the World Health Organization (WHO) classify carbapenemase‑producing Enterobacteriaceae (CPE) as urgent antimicrobial‑resistance threats, underscoring the high‑risk nature of critical‑care settings (2).

Imipenem, a broad‑spectrum carbapenem resistant to most β‑lactamases, remains a cornerstone for treating severe Gram‑negative infections. However, the emergence of imipenem‑resistant Enterobacterales has compromised this last‑line defense, correlating with increased morbidity and mortality. Infections caused by Klebsiella pneumoniae carbapenemase (KPC)‑producing Enterobacter species have shown mortality rates up to 33% compared to 6% in imipenem‑susceptible cases (3).

At the molecular level, imipenem resistance in E. coli is predominantly mediated by the acquisition or expression of carbapenemases enzymes that hydrolyze carbapenems and other β‑lactams. Key carbapenemase gene families include blaNDM, blaOXA‑48‑like, blaKPC, blaVIM, and blaIMP, all widely disseminated among diverse E. coli lineages, including sequence type 131 (ST131) (4). Resistance may be further enhanced by mechanisms such as loss or alteration of outer membrane porins (e.g., OmpC, OmpF) and co‑production of extended‑spectrum β‑lactamases (ESBLs) or AmpC β‑lactamases, which can collectively elevate imipenem minimum inhibitory concentrations (MICs) to clinically resistant levels (5).

While numerous studies have explored carbapenem resistance across Enterobacteriaceae, most reviews adopt a broad focus, spanning multiple species, wards, or community settings, without zeroing in on imipenem resistance in E. coli from ICU/NICU environments (6). Such specificity is crucial for informing antibiotic stewardship, refining infection‑control protocols, and guiding molecular surveillance in the most vulnerable patient populations. This systematic review and meta‑analysis aims to (i) estimate the pooled prevalence of imipenem resistance among E. coli isolates from ICU settings; and (ii) assess temporal, geographic, and methodological factors contributing to heterogeneity across studies.

# Methods

This systematic review and meta-analysis was conducted and reported in accordance with the PRISMA 2020 statement. The protocol-defined methodological domains included eligibility criteria, information sources, search strategy, study selection, data extraction, variables collected, risk-of-bias assessment, outcome measures, and statistical synthesis.

**Eligibility criteria**

We included observational studies reporting imipenem resistance among **clinical** Escherichia coli isolates obtained from **ICU and/or NICU settings,** including surveillance studies, cross-sectional studies, and cohort-like case series with adequate isolate counts. Eligible studies were required to: (i) evaluate imipenem resistance in E. coli from ICU/NICU populations; (ii) report, or allow derivation of, the number of imipenem-resistant isolates and the total number of E. coli isolates tested; and (iii) be available as full-text articles in English.

We excluded non-English publications, case reports, very small case series, pharmacokinetic/pharmacodynamic studies, reviews, editorials, letters, outbreak descriptions without extractable denominators, non-human studies, and studies from mixed hospital settings when ICU/NICU-specific data could not be separated. When multiple publications appeared to include overlapping populations or study periods, only the most comprehensive non-duplicative dataset was retained.

**Information sources and search strategy**

A systematic literature search was conducted in **PubMed/MEDLINE, EMBASE, Scopus, and Web of Science** from database inception to **1 June 2025**. Reference lists of included articles and relevant reviews were also screened to identify additional eligible reports. No language restriction was applied during database searching; the English-language criterion was applied during full-text eligibility assessment.

The core search strategy combined terms for the organism, antibiotic class/drug, resistance phenotype, and critical care setting, as follows:

(“Escherichia coli”[MeSH Terms] OR “Escherichia coli” OR E. coli) AND (imipenem OR carbapenem) AND (resistan OR non-suscept* OR nonsuscept*) AND (ICU OR “intensive care” OR NICU OR “neonatal intensive”)**.

Search syntax was adapted as needed for each database.

**Selection process**

All retrieved records were imported into **EndNote version 20** and duplicates were removed. Screening was performed in two sequential stages: title/abstract screening followed by full-text assessment. Two reviewers independently assessed records at each stage, and disagreements were resolved by discussion; unresolved discrepancies were adjudicated by a third reviewer. Reasons for exclusion at the full-text stage were documented. The study selection process is presented in the PRISMA 2020 flow diagram.

#### Data collection process

Data extraction was performed independently by two reviewers using a piloted standardized extraction form. Discrepancies were resolved by consensus or by third-reviewer adjudication. When imipenem-specific numerators or denominators were incomplete or unclear, corresponding authors were contacted where feasible.

#### Data items

The following study-level variables were extracted: first author, publication year, country, WHO region, continent, development status, World Bank income category, study setting (ICU, NICU, or mixed critical care setting with extractable ICU/NICU data), study period, year group, decade, specimen/source of isolates, and infection versus colonization status where reported. These variables were collected to enable investigation of the global distribution, regional frequency, and temporal pattern of imipenem resistance across different epidemiologic settings.

Laboratory-related variables included antimicrobial susceptibility testing (AST) method, categorized as disc diffusion, broth microdilution, automated system, or other/unknown, and interpretive guideline, categorized as CLSI, EUCAST, multiple standards, or unspecified/other. Where available, breakpoint version/year was also extracted to examine methodological variation in reported resistance frequencies.

Specimen/source of isolates and infection versus colonization status were extracted where explicitly reported; however, these variables were incompletely reported and were therefore not suitable for formal subgroup meta-analysis.

The primary outcome extracted for quantitative synthesis was the number of imipenem-resistant *E. coli* isolates and the total number of *E. coli* isolates tested (N), from which the study-specific resistance proportion (R/N) was calculated. For planned subgroup analyses, data were additionally organized according to country, WHO region, continent, income category, development status, study period, year group, decade, ICU versus NICU setting, specimen source, infection versus colonization status, AST method, and AST guideline. Where available, secondary molecular data, including the frequency of major carbapenemase genes and the methods used for their detection, were also extracted.

#### Risk-of-bias assessment

Risk of bias was assessed using the **Joanna Briggs Institute (JBI) Critical Appraisal Checklist for Studies Reporting Prevalence Data**. Two reviewers evaluated each study independently, and disagreements were resolved by a third reviewer. Item-level assessments and overall study judgments were tabulated.

#### Effect measure

The primary effect measure was the **study-specific proportion of imipenem resistance**, calculated as the number of resistant isolates divided by the total number of tested E. coli isolates (**R/N**).

Statistical synthesis

Meta-analysis of proportions was performed in **R version 4.2.1** using the **meta, metafor**, and **metasens** packages. For pooled estimation, the primary synthesis used a **random-effects model** with inverse-variance weighting as implemented in metaprop, and proportions were stabilized using the **Freeman–Tukey double arcsine transformation (PFT)**. Pooled estimates were back-transformed for presentation as proportions with **95% confidence intervals**. In additional model-based analyses, study-specific proportions were also transformed using the **logit transformation** (measure = "PLO") and fitted using rma in the **metafor** package with the **DerSimonian–Laird estimator** for between-study variance.

Heterogeneity was quantified using **τ², Cochran’s Q**, and **I²**. Forest plots were generated for the overall pooled prevalence and subgroup summaries. Subgroup analyses were prespecified according to **country, WHO region, continent, development status, income level, AST method, AST guideline, year group (1996–2016 vs 2017–2024), and decade (1990s, 2000s, 2010s, 2020s)**. Between-subgroup differences were examined using moderator models.

Meta-regression

To evaluate temporal change in resistance prevalence, random-effects meta-regression was conducted with **study year** as a continuous moderator. Model coefficients, 95% confidence intervals, and p values were used to assess the direction and significance of time trends. Bubble plots with model-based predicted values and confidence limits were generated to visualize temporal trajectories.

# Influence and outlier diagnostics

Potential outliers and influential studies were examined using **studentized residuals and Cook’s distances** from the fitted random-effects models. Studies with studentized residuals exceeding the **Bonferroni-adjusted threshold**, defined as the 100 × (1 − 0.05/(2k))th percentile of the standard normal distribution, were flagged as potential outliers. Studies with Cook’s distance greater than **median + 6 × IQR** were considered potentially influential. These diagnostics were used to assess whether pooled estimates were unduly driven by single studies.

# Assessment of reporting bias and small-study effects

Small-study effects and possible reporting bias were assessed using multiple complementary approaches. Conventional funnel-plot asymmetry was evaluated by the **rank correlation test (Begg and Mazumdar)** and **regression test (Egger-type test using standard error as predictor)**. In addition, **trim-and-fill analysis** was applied to estimate the potential impact of missing studies on the pooled prevalence. Because asymmetry tests based on funnel plots may be unstable in proportion meta-analysis, **Doi plots and the Luis Furuya–Kanamori (LFK) index** were also generated using the **metasens** package. LFK values were interpreted conventionally as indicating **no asymmetry** (absolute value <1), **minor asymmetry** (1 to 2), or **major asymmetry** (>2). A **Fail-safe N** was also calculated as a supplementary robustness metric.

# Software and reproducibility

All analyses and figures were generated in **R 4.2.1** with the packages used in the analytic workflow, including **metafor**, **meta**, **metasens**, **tidyverse**, and supporting visualization packages. The analytic script implemented automated generation of pooled estimates, subgroup analyses, temporal meta-regression, influence diagnostics, DOI plots with LFK index, funnel plots, trim-and-fill analyses, and summary outputs in tabular and graphical form.

# Results

# Search Results and Study Characteristics

The systematic search of PubMed/MEDLINE, EMBASE, Scopus, and Web of Science identified 752 records. After removal of duplicates and screening of titles and abstracts, 638 records were excluded. The remaining 114 articles underwent full-text assessment for eligibility. Of these, 72 studies were excluded because they were reviews or meta-analyses, conference abstracts without full text, included adult populations or non-invasive diseases, or did not provide relevant data. Consequently, 42 studies met the eligibility criteria and were included in both the qualitative and quantitative syntheses (Figure 1). The characteristics and key findings of all included studies are summarized in Table 1.

Overall Pooled Prevalence

Across 42 studies including 15,227 ICU E. coli isolates, 752 were imipenem resistant, yielding a pooled prevalence of 0.095 (95% CI 0.056–0.157) under a random-effects model (Figure 2). Between-study heterogeneity was high (Q(41) = 1462.276, p < 0.001; I² = 97.2%; τ² = 3.1662). The 95% prediction interval was 0.005 to 0.40, indicating the expected range of prevalence in a future similar study under the observed heterogeneity. A trim-and-fill analysis produced an adjusted pooled estimate of 0.103 (95% CI 0.060–0.170), indicating minimal shift in the central estimate (Table 2).

# Influence Diagnostics

No study exceeded the Bonferroni‑adjusted studentized residual threshold (maximum observed = **3.241**), and Cook’s distances did not flag any overly influential studies. These findings suggest that the pooled estimate is not driven by single‑study effects (**Figure 2**; **Table 2**).

# Integrated Subgroup Analyses

To synthesize subgroup findings efficiently, we report contrasts by geography, laboratory methodology, and time, with corresponding pooled proportions, 95% CIs, and heterogeneity metrics.

**Geography (countries):** Resistance varied markedly across countries (**P** for between‑country differences = **0.036**). The **United States** showed the lowest prevalence (**0.5%**, 1 report; 17/3753), while **Peru** showed the highest (**85.7%**, 1 report; 6/7). Among multi‑study countries: **China** pooled at **4.7%** (7 reports; 273/7180; **I²** 92.25%), **Taiwan** at **4.8%** (4; 69/807; **I²** 96.01%), **Turkey** at **9.5%** (6; 57/784; **I²** 95.80%), **Iran** at **11.9%** (6; 35/302; **I²** 81.44%), **Saudi Arabia** at **18.2%** (3; 18/100; **I²** 0.00%), **Bangladesh** at **33.3%** (1; 3/9), and **India** at **39.7%** (5; 198/1329; **I²** 98.23%). **Romania** pooled at **12.6%** (2; 5/41; **I²** 74.68%), and **Egypt** at **18.6%** (1; 32/172). These country‑level estimates align with the visual geospatial gradients shown in **Figure 3A**.

**WHO region and continents:** By WHO region (**P** = **0.031**), prevalence was lowest in the **Western Pacific** (**4.6%**, 11 reports; 342/7987; **I²** 96.13%) and highest in **South‑East Asia** (**38.4%**, 6; 201/1338; **I²** 97.80%). The **Eastern Mediterranean** pooled at **17.3%** (12; 119/650; **I²** 85.55%), the **European Region** at **10.0%** (8; 62/825; **I²** 94.31%), and the **Region of the Americas** at **13.3%** (2; 23/3760; **I²** 97.63%). By continent (not statistically significant; **P** = **0.985**), **Asia** pooled at **11.2%** (34; 687/10,587; **I²** 97.29%), **Europe** at **12.6%** (2; 5/41; **I²** 74.68%), **Africa** at **18.6%** (1; 32/172), and the **Americas** at **13.3%** (2; 23/3760; **I²** 97.63%).

**Socio‑economic context:** Development status did not significantly modify prevalence (**P** = **0.622**): **developing** settings pooled at **13.2%** (29 reports; 638/9859; **I²** 97.55%) versus **developed** at **9.1%** (6; 40/3894; **I²** 96.38%). By **income level** (**P** = **0.043**), **lower‑middle‑income** contexts had the highest prevalence (**32.5%**, 9; 267/1586; **I²** 97.19%), while **upper‑middle‑income** settings had the lowest (**8.4%**, 20; 371/8273; **I²** 94.58%); **high‑income** pooled at **9.1%** (6; 40/3894; **I²** 96.38%).

**Laboratory methodology:** Prevalence differed by AST method (**P** = **0.005**). **Broth microdilution** yielded the lowest pooled estimate (**4.6%**, 19; 418/12,908; **I²** 96.80%), followed by **disc diffusion (Kirby–Bauer)** (**19.7%**, 17; 307/1952; **I²** 95.54%) and **automated systems** (**19.3%**, 5; 26/150; **I²** 39.85%). Studies categorized as **Other/Unknown** method pooled at **0.5%** (1; 1/217). By AST guideline, **CLSI**‑based studies pooled at **8.4%** (38; 680/14,868; **I²** 97.29%), **EUCAST** at **46.2%** (1; 6/13), and **mixed CLSI+EUCAST** at **22.3%** (3; 66/346; **I²** 93.11%). The EUCAST subgroup derives from a single, small study and should be interpreted with considerable caution.

**Time period:** Imipenem resistance increased over time. By year group (**P** = **0.034**), prevalence rose from **5.1%** in **1996–2016** (20 reports; 199/7395; **I²** 96.44%) to **16.3%** in **2017–2024** (22; 553/7832; **I²** 97.73%). By decade (**P** = **0.088**), pooled prevalence was **1.5%** in the **1990s** (4; 13/779; **I²** 81.27%), **4.4%** in the **2000s** (3; 57/4101; **I²** 99.24%), **9.1%** in the **2010s** (20; 173/2918; **I²** 93.85%), and **18.0%** in the **2020s** (15; 509/7429; **I²** 98.28%).

## Temporal Trend (Meta‑Regression)

Bubble meta‑regression demonstrated a statistically significant upward trend in imipenem resistance over calendar time (**r = 0.113**, **p = 0.005**, 95% CI **0.034–0.192**), with model‑based predictions shown in **Figure 4**.

## Geospatial Patterns

Country‑level mapping (**Figure 3B**) emphasizes very low pooled prevalence in the **USA** (0.5%) and **Taiwan/China** (≈5%), intermediate prevalence across parts of the **Eastern Mediterranean** (e.g., **Iran** 11.9%, **Turkey** 9.5%, **Saudi Arabia** 18.2%, **Egypt** 18.6%), and high prevalence in **South‑East Asia** (e.g., **India** 39.7%, **Bangladesh** 33.3%). The single study from **Peru** plotted at **85.7%** underscores the influence of small denominators and warrants cautious interpretation.

## Small‑Study Effects and Reporting Bias

The Doi plot indicated **major asymmetry** with **LFK index = 4.84** (**Figure 5A**), whereas conventional tests did not detect funnel asymmetry (Begg **p = 0.651**; Egger **p = 0.867**) (**Figure 5B**; **Table 2**). The **Fail‑Safe N = 21,541** suggests robustness of the overall finding to potential unpublished null studies. Trim‑and‑fill imputation shifted the pooled estimate only slightly (to **10.3%**, 95% CI **6.0–17.0%**), supporting stability of the central tendency despite small‑study effects.

# Pooled Prevalence of Carbapenemase Genes

The pooled prevalence of carbapenemase genes showed substantial variation across the included studies (Figure 6). *bla*_NDM_ was the most frequently detected carbapenemase gene, with a pooled prevalence of approximately 83%, followed by *bla*_OXA-48_-like at approximately 74% and *bla*_KPC_ at approximately 45%. Lower pooled frequencies were observed for *bla*_VIM_ and *bla*_IMP_, while several other carbapenemase genes were detected at substantially lower frequencies. Overall, the distribution demonstrated a predominance of *bla*_NDM_ and *bla*_OXA-48_-like, with considerable heterogeneity in the reported prevalence of individual carbapenemase determinants across studies (Figure 6).

**Discussion**

The pooled prevalence of imipenem resistance among *E. coli* isolates from intensive care units (ICUs) was 9.5% (95% CI 5.6–15.7), highlighting the clinical challenge posed by last-line β-lactams in critical-care settings.

This pooled estimate should be interpreted as a global epidemiologic benchmark rather than a universal threshold for empirical treatment decisions. In practice, ICU antimicrobial selection should remain guided by local antibiograms, patient severity, infection source, and contemporaneous susceptibility data. This estimate is lower than the 20.6% overall prevalence reported in a Chinese tertiary-care cohort of gastrointestinal fistula patients (7), suggesting that site-specific selection pressures and patient populations significantly influence resistance burden. The negligible change in the pooled estimate after trim-and-fill adjustment (from 9.5% to 10.3%) indicates that unpublished studies with null findings are unlikely to overturn the central tendency, enhancing confidence in the meta-analytic result. (8). Nevertheless, the high between-study heterogeneity (I² = 97.2%) signals substantial variability in resistance prevalence across geographic regions, temporal epochs, and methodological frameworks, prompting a need to dissect contributory factors (9). Together, these observations highlight the importance of context-tailored antimicrobial stewardship strategies to mitigate imipenem resistance in vulnerable ICU/NICU populations.

Sources of heterogeneity in imipenem-resistance estimates can be traced to variations in regional antimicrobial consumption, diagnostic methodologies, and health-system attributes. High antibiotic pressure in ICUs characterized by broad-spectrum β-lactam use and prolonged device utilization has been implicated in the selection of carbapenem-resistant pathogens. (10). In lower-middle-income settings, this pressure is compounded by limited infection-control infrastructure, driving the emergence and persistence of resistant clones (11). Diagnostic heterogeneity further amplifies variability, as laboratories employing distinct antimicrobial susceptibility testing (AST) methods and interpretive guidelines may classify marginal MIC elevations differently, leading to discordant resistance reports (12). These intertwined factors collectively contribute to the observed variability in imipenem-resistance prevalence and underscore the necessity of harmonizing laboratory practices alongside stewardship interventions (12).

Influence diagnostics confirmed the robustness of the pooled prevalence estimate, with no individual study exerting undue leverage on the meta-analytic model. The absence of Bonferroni-adjusted outliers and modest Cook’s distances suggest that the 9.5% estimate is not artifactually driven by atypical cohorts or extreme findings but reflects a genuine epidemiological signal (13). This stability across studies deploying diverse designs, including cross-sectional surveys, prospective cohorts, and retrospective audits, lends weight to the validity of the synthesized measure (12). Consequently, policy directives and clinical guidelines based on this estimate can be promulgated with reduced concern for single-study bias. However, vigilance for emerging outlier data remains prudent, particularly in rapidly evolving antimicrobial landscapes (13).

Subgroup analyses by country revealed starkly contrasting imipenem-resistance burdens, with the United States exhibiting a low prevalence of 0.5% compared to 85.7% in a small Peruvian cohort, illustrating the influence of both large denominators and stewardship maturity (11). Multi-study nations fell along a spectrum: China (4.7%), Taiwan (4.8%), Turkey (9.5%), Iran (11.9%), Saudi Arabia (18.2%), Bangladesh (33.3%), and India (39.7%), mirroring regional gradients in antimicrobial regulation and hospital-level infection control (Liu et al., 2017). The Western Pacific region, encompassing China and Taiwan, showed the lowest pooled resistance (4.6%), while South-East Asia, driven by data from Bangladesh and India, peaked at 38.4%, underscoring the impact of resource limitations on antibiotic stewardship (8). These geographic disparities emphasize that national and regional policies must be calibrated to local epidemiology rather than extrapolated from global averages (8).

Analysis by WHO region reinforced country-level observations, with the Western Pacific region exhibiting the lowest imipenem resistance (4.6%) and South-East Asia the highest (38.4%), while the Eastern Mediterranean, European, and American regions occupied intermediate positions (17.3%, 10.0%, and 13.3%, respectively) (8). Continental stratification yielded qualitatively similar trends, although statistical significance was attenuated (P = 0.985), reflecting the uneven distribution of ICU data across continents (7). Importantly, these findings align with global surveillance reports that identify South-East Asia as a hotspot for carbapenem-resistant Enterobacterales, driven by unregulated antibiotic access and overcrowded healthcare settings (8). Consequently, prioritization of stewardship resources and infection-control training in high-burden regions is imperative.

Socio-economic context modified resistance prevalence, with lower-middle-income settings showing the highest pooled estimate (32.5%) compared to high-income (9.1%) and upper-middle-income (8.4%) contexts (11). Although the contrast between developing (13.2%) and developed (9.1%) settings was not statistically significant (P = 0.622), the higher imipenem-resistance rates in resource-constrained health systems likely reflect gaps in antimicrobial stewardship programs and laboratory capacity (11). Resource limitations also hinder prompt detection and reporting of carbapenem resistance, perpetuating transmission within ICU environments (12). Strengthening healthcare infrastructure and ensuring a consistent supply of AST reagents are therefore foundational to mitigating imipenem resistance in low-resource settings.

Laboratory methodology emerged as a significant determinant of reported resistance prevalence (P = 0.005), with broth microdilution yielding a markedly lower pooled estimate (4.6%) compared with disc diffusion (19.7%) and automated systems (19.3%) (13). This pattern is consistent with the higher analytic precision and standardization of broth microdilution, the reference method for phenotypic susceptibility testing, whereas diffusion-based and automated platforms rely on categorical interpretations that may be more vulnerable to breakpoint-version changes, inter-laboratory variability, and misclassification near susceptibility thresholds (13). However, these differences may also reflect confounding by study geography, period of data collection, and laboratory resource level, as facilities employing broth microdilution often differ systematically from those using other AST methods. Notably, studies reporting mixed or unspecified methodologies showed implausibly low prevalence estimates (0.5%), highlighting that incomplete methodological reporting can distort pooled analyses (12). Strengthening methodological transparency, harmonizing AST protocols, and ensuring routine participation in external quality-assurance programs remain essential to improve comparability of resistance estimates across settings (12).

Interpretive guidelines influenced the pooled prevalence estimates, with CLSI-based studies reporting 8.4% resistance, EUCAST-based studies 46.2%, and mixed CLSI+EUCAST classifications 22.3% (12). EUCAST’s generally lower breakpoints may increase sensitivity for detecting carbapenem resistance, potentially elevating the apparent resistance rates compared to CLSI criteria (12). The presence of mixed-guideline reporting further complicates data synthesis and may contribute to increased heterogeneity across studies (12). Adoption of a standardized, globally endorsed interpretive framework, regularly updated to reflect emerging evidence, would enhance the consistency and reliability of resistance surveillance in ICU settings.

However, the apparent discrepancy between CLSI- and EUCAST-based prevalence should be interpreted with caution, as the EUCAST subgroup is represented by only a single small study. While breakpoint differences between these guidelines can influence resistance categorization, the primary cause of instability in this comparison is the limited EUCAST data.

Temporal stratification indicated a clear upward trend in imipenem resistance over the past three decades, rising from 1.5% in the 1990s to 18.0% in the 2020s (P = 0.088 by decade), and from 5.1% in 1996–2016 to 16.3% in 2017–2024 (P = 0.034) (7, 8). These trends mirror global reports of escalating carbapenem use in critical-care environments (7, 8). Bubble meta-regression confirmed a statistically significant upward slope (r = 0.113 per year; p = 0.005), projecting continued resistance escalation if current stewardship practices remain unchanged (7). The temporal acceleration of imipenem resistance highlights an urgent need for dynamic antibiotic-use policies and real-time resistance monitoring.

Meta-regression findings offer actionable insights for policymakers: each calendar year is associated with a quantifiable increase in resistance prevalence, suggesting that improvements in antibiotic stewardship could decelerate the upward trend (7, 8). Simulation of stewardship-induced breakpoint shifts and consumption reductions may inform cost-effectiveness analyses, guiding resource allocation toward interventions with maximal epidemiological impact (7). Moreover, regionally tailored stewardship bundles that integrate prescriber education and rapid diagnostic support have demonstrated efficacy in reducing carbapenem resistance in ICU settings (9, 14).

Geospatial mapping elucidated global hotspots of imipenem resistance: very low prevalence in the USA (0.5%), intermediate levels in the Eastern Mediterranean (9.5–18.6%), and high burdens in South-East Asia (33–40%) (11). The outlier 85.7% estimate from Peru, derived from a small sample size, exemplifies the distortion possible when sparse data are extrapolated without caution (11). These spatial patterns should inform the prioritized deployment of infection-control resources and stewardship training to regions bearing the greatest resistance burden (11).

Assessment of small-study effects indicated potential reporting bias among smaller studies, despite conventional funnel tests not detecting asymmetry (13). The large Fail-Safe N (21,541) indicates that many unpublished null results would be needed to overturn the pooled estimate, supporting its overall robustness (13). Nevertheless, continued incentivization of negative and null-result publication is crucial to mitigate selective reporting and ensure a balanced evidence base (Lutgring et al., 2023).

The high prevalence and accelerating trend of imipenem resistance in ICU/NICU E. coli isolates have important clinical implications. As imipenem represents a core component of empirical therapy for severe Gram-negative sepsis, rising resistance threatens to erode last-line treatment efficacy, potentially increasing morbidity, length of stay, and healthcare costs (Liu et al., 2017), Yang et al., 2019). Clinicians should be aware of local resistance patterns and may need to adjust empirical regimens based on susceptibility results (Meyer et al., 2010; , Akazawa et al., 2019). Simultaneously, hospitals must reinforce infection-control measures to curb intra-unit transmission of resistant clones (9, 10).

Limitations of this meta-analysis warrant careful consideration. The high between-study heterogeneity limits the precision of subgroup estimates and reflects the influence of unmeasured confounders such as local prescribing habits and patient-case mix (7). Variation in laboratory methodology and interpretive guidelines complicates data synthesis, and although subgroup analyses partially addressed these factors, residual inconsistencies likely persist (12). Geographic data gaps, especially from Latin America and sub-Saharan Africa, may bias pooled estimates toward better-studied regions (11). Finally, the cross-sectional nature of included studies precludes causal inference regarding stewardship interventions and resistance trends.

Future research should prioritize longitudinal, multicenter surveillance of ICU/NICU E. coli isolates employing standardized AST methods and unified interpretive criteria to produce high-fidelity resistance data. Molecular characterization of resistance mechanisms particularly carbapenemase gene carriage should be integrated into routine surveillance to track clonal expansion and horizontal gene transfer (15, 16). Deployment of rapid phenotypic assays can accelerate detection of carbapenemase producers (16). Furthermore, coupling resistance surveillance with antimicrobial-consumption metrics will enable robust evaluation of stewardship interventions and identification of best practices for reducing imipenem resistance in critical-care settings (9, 14).

In summary, this meta-analysis provides a comprehensive estimate of imipenem resistance among ICU/NICU *E. coli* isolates (9.5%, 95% CI 5.6–15.7) and reveals significant geographic, socio-economic, methodological, and temporal heterogeneity. The upward trend in resistance underscores an urgent need for concerted global efforts in laboratory standardization, antimicrobial stewardship, infection control, and molecular surveillance to safeguard the efficacy of last-line carbapenems.

## Conclusions

Imipenem resistance among E. coli isolates from ICU/NICU settings is substantial affecting roughly one in ten isolates worldwide and shows pronounced geographic, economic, and methodological heterogeneity alongside a significant upward temporal trend. Higher prevalence in lower-middle-income regions and variability across AST methods highlight gaps in laboratory harmonization and surveillance. These findings emphasize the urgent need for strengthened antimicrobial stewardship, standardized susceptibility testing, and expanded molecular monitoring particularly for blaNDM and OXA-48–like carbapenemases to inform empiric therapy and curb further spread in critical care environments.

## Declarations

**Ethics approval**: Not applicable (systematic review).

**Consent for publication**: Not applicable.

**Competing interests**: The authors declare no competing interests.

**Funding**: Not applicable.

**Authors’ contributions:**
SBP conceptualized and designed the study, performed the literature search and data extraction, conducted the statistical analyses, and drafted the initial manuscript. RN contributed to study design, data interpretation, critical revision of the manuscript for important intellectual content, and supervised the research process. SSGK contributed to methodology development, quality assessment and risk of bias evaluation, data interpretation, and critically reviewed and edited the manuscript. All authors read and approved the final manuscript and agree to be accountable for all aspects of the work.

**Figure 1.** PRISMA 2020 flow diagram of study selection.

**Figure 2**: Overall Worldwide Forest plot for pooled imipenem resistance prevalence of E. coli in ICU/NICU. The boxplots represent the distribution of resistance proportions (%) across studies, with individual data points shown as jittered dots. The red squares indicate the pooled resistance proportion for each antibiotic, with error bars representing the 95% confidence intervals (CI). The leftmost column shows the number of studies (K), resistant isolates (R), and total isolates (N). The rightmost columns display the heterogeneity metric (I²) and its corresponding p-value (P1).

**Figure 3.** Subgroup forest plots by setting and region.

**Figure 4**: Meta-regression analysis results were illustrated in scatter plot of trend of proportion of Imipenem-resistant isolates during years.

**Figure 5.** **A**: Doi plots; **B**: Funnel plot, fill and trim method.

**Figure 6.** Pooled prevalence of carbapenemase genes (*blaNDM*, *blaOXA*-48-like, *blaKPC*, *blaVIM*, *blaIMP*).

**Table 1.** Characteristics of included studies (country, period, setting, isolates, AST method, breakpoints, ICU/NICU, infection vs colonization, specimen, outcomes reported).

**Table 1**: Characteristics and Extracted Data of Studies Included in the Meta-analysis

| author | Year | Country | Continent | Who_region | Development_status | Income_level | AST_method | AST_guideline | Year_group | Decade | Total | Imipenem |
| --- | --- | --- | --- | --- | --- | --- | --- | --- | --- | --- | --- | --- |
| Nipa Singh Et Al | 2016 | India | Asia | South-East Asia Region | Developing | Lower middle income | Disc diffusion (Kirby–Bauer) | CLSI | 1996_2016 | 2010s | 1038 | 9 |
| Abid Hussain Chang Et Al | 2013 | Pakistan | Asia | Eastern Mediterranean Region | Developing | Lower middle income | Disc diffusion (Kirby–Bauer) | CLSI | 1996_2016 | 2010s | 27 | 2 |
| Fan Chang Et Al | 2023 | China | Asia | Western Pacific Region | Developing | Upper middle income | Broth microdilution | CLSI | 2017_2024 | 2020s | 5684 | 194 |
| Nima Davari Et Al | 2022 | Iran | Asia | Eastern Mediterranean Region | Developing | Upper middle income | Disc diffusion (Kirby–Bauer) | CLSI | 2017_2024 | 2020s | 25 | 2 |
| David García-Cedró Et Al | 2023 | Peru | Americas | Region of the Americas | Developing | Upper middle income | Broth microdilution | CLSI | 2017_2024 | 2020s | 7 | 6 |
| Garima Gautam Et Al | 2024 | India | Asia | South-East Asia Region | Developing | Lower middle income | Disc diffusion (Kirby–Bauer) | CLSI | 2017_2024 | 2020s | 226 | 147 |
| Y Glupczynski Et Al | 1998 | NA | NA | NA | NA | NA | Broth microdilution | CLSI | 1996_2016 | 1990s | 285 | 2 |
| Rifat Yasmin Et Al | 2022 | Pakistan | Asia | Eastern Mediterranean Region | Developing | Lower middle income | Disc diffusion (Kirby–Bauer) | CLSI | 2017_2024 | 2020s | 49 | 32 |
| M Mohammadi-Mehr Et Al | 2011 | Iran | Asia | Eastern Mediterranean Region | Developing | Upper middle income | Disc diffusion (Kirby–Bauer) | CLSI | 1996_2016 | 2010s | 60 | 1 |
| Harun Ağca Et Al | 2011 | Turkey | Asia | European Region | Developing | Upper middle income | Disc diffusion (Kirby–Bauer) | CLSI | 1996_2016 | 2010s | 47 | 3 |
| Shadi Aghamohammad Et Al | 2019 | Iran | Asia | Eastern Mediterranean Region | Developing | Upper middle income | Disc diffusion (Kirby–Bauer) | CLSI | 2017_2024 | 2010s | 61 | 2 |
| Ruoming Tan Et Al | 2014 | China | Asia | Western Pacific Region | Developing | Upper middle income | Other/Unknown | CLSI | 1996_2016 | 2010s | 217 | 1 |
| Ahmad Alikhani Et Al | 2012 | Iran | Asia | Eastern Mediterranean Region | Developing | Upper middle income | Broth microdilution | EUCAST | 1996_2016 | 2010s | 13 | 6 |
| Anushya Annamalai Et Al | 2020 | India | Asia | South-East Asia Region | Developing | Lower middle income | Disc diffusion (Kirby–Bauer) | CLSI | 2017_2024 | 2020s | 3 | 1 |
| Nahla Shazli Abdel Azim Et Al | 2019 | Saudi Arabia | Asia | Eastern Mediterranean Region | Developed | High income | Disc diffusion (Kirby–Bauer) | CLSI | 2017_2024 | 2010s | 16 | 4 |
| Abdulrahman S Bazaid Et Al | 2022 | Saudi Arabia | Asia | Eastern Mediterranean Region | Developed | High income | Automated system | CLSI | 2017_2024 | 2020s | 21 | 4 |
| Ruiqi Xiao Et Al | 2023 | China | Asia | Western Pacific Region | Developing | Upper middle income | Broth microdilution | CLSI | 2017_2024 | 2020s | 370 | 7 |
| Hadi Hamishehkar Et Al | 2016 | Iran | Asia | Eastern Mediterranean Region | Developing | Upper middle income | Disc diffusion (Kirby–Bauer) | CLSI | 1996_2016 | 2010s | 93 | 10 |
| Ayşe Betül Ergül Et Al | 2017 | Turkey | Asia | European Region | Developing | Upper middle income | Automated system | Multiple (CLSI + EUCAST) | 2017_2024 | 2010s | 4 | 2 |
| Chih-Cheng Lai Et Al | 2019 | Taiwan | Asia | Western Pacific Region | NA | NA | Broth microdilution | CLSI | 2017_2024 | 2010s | 121 | 1 |
| M. Yücesoy Et Al | 2013 | Turkey | Asia | European Region | Developing | Upper middle income | Broth microdilution | CLSI | 1996_2016 | 2010s | 273 | 4 |
| Noha Alaa Eldin Fahim Et Al | 2021 | Egypt | Africa | Eastern Mediterranean Region | Developing | Lower middle income | Disc diffusion (Kirby–Bauer) | CLSI | 2017_2024 | 2020s | 172 | 32 |
| Kai Yin Et Al | 2022 | China | Asia | Western Pacific Region | Developing | Upper middle income | Automated system | CLSI | 2017_2024 | 2020s | 35 | 9 |
| Zohreh Mohammadtaheri Et Al | 2010 | Iran | Asia | Eastern Mediterranean Region | Developing | Upper middle income | Disc diffusion (Kirby–Bauer) | CLSI | 1996_2016 | 2010s | 50 | 14 |
| Shio-Shin Jean Et Al | 2015 | Taiwan | Asia | Western Pacific Region | NA | NA | Broth microdilution | CLSI | 1996_2016 | 2010s | 344 | 4 |
| Tanzina Nusrat Et Al | 2019 | Bangladesh | Asia | South-East Asia Region | Developing | Lower middle income | Disc diffusion (Kirby–Bauer) | CLSI | 2017_2024 | 2010s | 9 | 3 |
| Ying Ding, Et Al | 2023 | China | Asia | Western Pacific Region | Developing | Upper middle income | Broth microdilution | CLSI | 2017_2024 | 2020s | 214 | 16 |
| Alice Elena Ghenea Et Al | 2022 | Romania | Europe | European Region | Developed | High income | Disc diffusion (Kirby–Bauer) | CLSI | 2017_2024 | 2020s | 14 | 4 |
| Andreea-Loredana Golli Et Al | 2022 | Romania | Europe | European Region | Developed | High income | Automated system | CLSI | 2017_2024 | 2020s | 27 | 1 |
| Filiz Günseren Et Al | 1999 | Turkey | Asia | European Region | Developing | Upper middle income | Broth microdilution | CLSI | 1996_2016 | 1990s | 112 | 8 |
| Qiwen Yang Et Al | 2020 | China | Asia | Western Pacific Region | Developing | Upper middle income | Broth microdilution | CLSI | 2017_2024 | 2020s | 519 | 44 |
| Y. Glupczynski Et Al | 1998 | NA | NA | NA | NA | NA | Broth microdilution | CLSI | 1996_2016 | 1990s | 258 | 2 |
| H Hanberger Et Al | 1999 | NA | NA | NA | NA | NA | Broth microdilution | CLSI | 1996_2016 | 1990s | 124 | 1 |
| Şaban İnceci̇k Et Al | 2009 | Turkey | Asia | European Region | Developing | Upper middle income | Broth microdilution | CLSI | 1996_2016 | 2000s | 64 | 35 |
| Ian Friedland Et Al | 2003 | United States | Americas | Region of the Americas | Developed | High income | Broth microdilution | CLSI | 1996_2016 | 2000s | 3753 | 17 |
| Altaf Bandy Et Al | 2021 | Saudi Arabia | Asia | Eastern Mediterranean Region | Developed | High income | Automated system | CLSI | 2017_2024 | 2020s | 63 | 10 |
| Vineeta Khare Et Al | 2017 | India | Asia | South-East Asia Region | Developing | Lower middle income | Disc diffusion (Kirby–Bauer) | CLSI | 2017_2024 | 2010s | 51 | 30 |
| Jinhong Xie Et Al | 2017 | China | Asia | Western Pacific Region | Developing | Upper middle income | Broth microdilution | CLSI | 2017_2024 | 2010s | 141 | 2 |
| Sanjith Saseedharan Et Al | 2016 | India | Asia | South-East Asia Region | Developing | Lower middle income | Disc diffusion (Kirby–Bauer) | CLSI | 1996_2016 | 2010s | 11 | 11 |
| H Leblebicioglu Et Al | 2002 | Turkey | Asia | European Region | Developing | Upper middle income | Broth microdilution | CLSI | 1996_2016 | 2000s | 284 | 5 |
| Shio-Shin Jean Et Al | 2015 | Taiwan | Asia | Western Pacific Region | NA | NA | Broth microdilution | Multiple (CLSI + EUCAST) | 1996_2016 | 2010s | 159 | 50 |
| Shio-Shin Jean Et Al | 2013 | Taiwan | Asia | Western Pacific Region | NA | NA | Broth microdilution | Multiple (CLSI + EUCAST) | 1996_2016 | 2010s | 183 | 14 |

Supplementary Table 2: Prevalence of Antibiotic Resistance

| Category | Subgroup | K (n, N) | Proportion  95%CI (LCI, HCI) | I² | P1 | P2 |
| --- | --- | --- | --- | --- | --- | --- |
| Overall | NA | 42 (752, 15227) | 0.095 (0.056, 0.157) | 97.20% | p<0.001 | NA |
| Country | India | 5 (198, 1329) | 0.397 (0.061, 0.871) | 98.23% | p<0.001 | p=0.036 |
|  | Pakistan | 2 (34, 76) | 0.294 (0.019, 0.902) | 93.68% | p<0.001 |  |
|  | China | 7 (273, 7180) | 0.047 (0.025, 0.087) | 92.25% | p<0.001 |  |
|  | Iran | 6 (35, 302) | 0.119 (0.048, 0.264) | 81.44% | p<0.001 |  |
|  | Peru | 1 (6, 7) | 0.857 (0.419, 0.980) | 0.00% | p>0.999 |  |
|  | Turkey | 6 (57, 784) | 0.095 (0.018, 0.373) | 95.80% | p<0.001 |  |
|  | Saudi Arabia | 3 (18, 100) | 0.182 (0.118, 0.271) | 0.00% | p=0.695 |  |
|  | Taiwan | 4 (69, 807) | 0.048 (0.009, 0.220) | 96.01% | p<0.001 |  |
|  | Egypt | 1 (32, 172) | 0.186 (0.135, 0.251) | 0.00% | p>0.999 |  |
|  | Bangladesh | 1 (3, 9) | 0.333 (0.111, 0.667) | 0.00% | p>0.999 |  |
|  | Romania | 2 (5, 41) | 0.126 (0.015, 0.583) | 74.68% | p=0.047 |  |
|  | United States | 1 (17, 3753) | 0.005 (0.003, 0.007) | 0.00% | p>0.999 |  |
| Continent | Asia | 34 (687, 10587) | 0.112 (0.064, 0.190) | 97.29% | p<0.001 | p=0.985 |
|  | Americas | 2 (23, 3760) | 0.133 (0.000, 0.994) | 97.63% | p<0.001 |  |
|  | Africa | 1 (32, 172) | 0.186 (0.135, 0.251) | 0.00% | p>0.999 |  |
|  | Europe | 2 (5, 41) | 0.126 (0.015, 0.583) | 74.68% | p=0.047 |  |
| WHO Region | South-East Asia Region | 6 (201, 1338) | 0.384 (0.075, 0.828) | 97.80% | p<0.001 | p=0.031 |
|  | Eastern Mediterranean Region | 12 (119, 650) | 0.173 (0.100, 0.283) | 85.55% | p<0.001 |  |
|  | Western Pacific Region | 11 (342, 7987) | 0.046 (0.023, 0.092) | 96.13% | p<0.001 |  |
|  | Region of the Americas | 2 (23, 3760) | 0.133 (0.000, 0.994) | 97.63% | p<0.001 |  |
|  | European Region | 8 (62, 825) | 0.100 (0.026, 0.315) | 94.31% | p<0.001 |  |
| Development Status | Developing | 29 (638, 9859) | 0.132 (0.071, 0.234) | 97.55% | p<0.001 | p=0.622 |
|  | Developed | 6 (40, 3894) | 0.091 (0.014, 0.407) | 96.38% | p<0.001 |  |
| Income Level | Lower middle income | 9 (267, 1586) | 0.325 (0.119, 0.633) | 97.19% | p<0.001 | p=0.043 |
|  | Upper middle income | 20 (371, 8273) | 0.084 (0.048, 0.145) | 94.58% | p<0.001 |  |
|  | High income | 6 (40, 3894) | 0.091 (0.014, 0.407) | 96.38% | p<0.001 |  |
| AST Method | Disc diffusion (Kirby–Bauer) | 17 (307, 1952) | 0.197 (0.094, 0.366) | 95.54% | p<0.001 | p=0.005 |
|  | Broth microdilution | 19 (418, 12908) | 0.046 (0.023, 0.089) | 96.80% | p<0.001 |  |
|  | Other/Unknown | 1 (1, 217) | 0.005 (0.001, 0.032) | 0.00% | p>0.999 |  |
|  | Automated system | 5 (26, 150) | 0.193 (0.114, 0.309) | 39.85% | p=0.156 |  |
| AST Guideline | CLSI | 38 (680, 14868) | 0.084 (0.047, 0.145) | 97.29% | p<0.001 | p=0.302 |
|  | EUCAST | 1 (6, 13) | 0.462 (0.224, 0.718) | 0.00% | p>0.999 |  |
|  | Multiple (CLSI + EUCAST) | 3 (66, 346) | 0.223 (0.064, 0.545) | 93.11% | p<0.001 |  |
| Year Group | 1996_2016 | 20 (199, 7395) | 0.051 (0.021, 0.119) | 96.44% | p<0.001 | p=0.034 |
|  | 2017_2024 | 22 (553, 7832) | 0.163 (0.081, 0.301) | 97.73% | p<0.001 |  |
| Decade | 2010s | 20 (173, 2918) | 0.091 (0.043, 0.182) | 93.85% | p<0.001 | p=0.088 |
|  | 2020s | 15 (509, 7429) | 0.180 (0.079, 0.362) | 98.28% | p<0.001 |  |
|  | 1990s | 4 (13, 779) | 0.015 (0.003, 0.063) | 81.27% | p=0.001 |  |
|  | 2000s | 3 (57, 4101) | 0.044 (0.001, 0.684) | 99.24% | p<0.001 |  |

**References**

(17-57)

## References

1.	Mohamed T, Yousef L, Darweesh E, Khalil AH, Meghezel ELz. Detection and Characterization of Carbapenem Resistant Enterobacteriacea in Sohag University Hospitals. Egyptian Journal of Medical Microbiology. 2018;27(4):61-9.

2.	Kotb M, Mowafy HL. Detection of Carbapenemase Producing Enterobacteriaceae Using the Modified Carbapenem Inactivation Method. Egyptian Journal of Medical Microbiology. 2019;28(4):171-7.

3.	Shin SY, Bae IK, Kim J, Jeong SH, Yong D, Kim JM, et al. Resistance to Carbapenems in Sequence Type 11 Klebsiella Pneumoniae Is Related to DHA-1 and Loss of OmpK35 and/or OmpK36. Journal of Medical Microbiology. 2012;61(2):239-45.

4.	Chang Y-T, Siu LK, Wang JT, Wu TL, Chen YH, Chuang Y-C, et al. &Lt;p&gt;Resistance Mechanisms and Molecular Epidemiology of Carbapenem-Nonsusceptible &Lt;em&gt;Escherichia Coli&lt;/Em&gt; In Taiwan, 2012-2015&lt;/P&gt. Infection and Drug Resistance. 2019;Volume 12:2113-23.

5.	Miao M, Wen H, Xu P, Niu S, Lv J, Xie X, et al. Genetic Diversity of Carbapenem-Resistant Enterobacteriaceae (CRE) Clinical Isolates From a Tertiary Hospital in Eastern China. Frontiers in Microbiology. 2019;9.

6.	Tsai Y-K, Liou CH, Fung CP, Lin JC, Siu LK. Single or in Combination Antimicrobial Resistance Mechanisms of Klebsiella Pneumoniae Contribute to Varied Susceptibility to Different Carbapenems. Plos One. 2013;8(11):e79640.

7.	Liu Q, Ren J, Wu X, Wang G, Wang Z, Wu J, et al. Shifting Trends in Bacteriology and Antimicrobial Resistance Among Gastrointestinal Fistula Patients in China: An Eight-Year Review in a Tertiary-Care Hospital. BMC Infectious Diseases. 2017;17(1).

8.	Yang S, Xu H, Sun J, Sun S. Shifting Trends and Age Distribution of ESKAPEEc Resistance in Bloodstream Infection, Southwest China, 2012–2017. Antimicrobial Resistance and Infection Control. 2019;8(1).

9.	Meyer E, Schwab F, Schroeren-Boersch B, Gastmeier P. Dramatic Increase of Third-Generation Cephalosporin-Resistant E. Coli in German Intensive Care Units: Secular Trends in Antibiotic Drug Use and Bacterial Resistance, 2001 to 2008. Critical Care. 2010;14(3).

10.	Yong D, Shin HB, Kim YK, Cho JH, Lee WG, Ha GY, et al. Increase in the Prevalence of Carbapenem-Resistant<i>Acinetobacter</I>Isolates and Ampicillin-Resistant Non-Typhoidal<i>Salmonella</I>Species in Korea: A KONSAR Study Conducted in 2011. Infection and Chemotherapy. 2014;46(2):84.

11.	Ramasubramanian V, Porwal R, Rajesh N. Carbapenem Resistant Gram-Negative Bacteremia in an Indian Intensive Care Unit: A Review of the Clinical Profile and Treatment Outcome of 50 Patients. Indian Journal of Critical Care Medicine. 2014;18(11):750-3.

12.	Sahu C, Jain V, Mishra P, Prasad KN. Clinical and Laboratory Standards Institute Versus European Committee for Antimicrobial Susceptibility Testing Guidelines for Interpretation of Carbapenem Antimicrobial Susceptibility Results for Escherichia Coli in Urinary Tract Infection (UTI). Journal of Laboratory Physicians. 2018;10(03):289-93.

13.	Lutgring JD, Kent AG, Bowers JR, Jasso‐Selles DE, Albrecht V, Stevens V, et al. Comparison of Carbapenem-Susceptible and Carbapenem-Resistant Enterobacterales at Nine Sites in the USA, 2013–2016: A Resource for Antimicrobial Resistance Investigators. Microbial Genomics. 2023;9(11).

14.	Akazawa T, Kusama Y, Fukuda H, Hayakawa K, Kutsuna S, Moriyama Y, et al. Eight-Year Experience of Antimicrobial Stewardship Program and the Trend of Carbapenem Use at a Tertiary Acute-Care Hospital in Japan—The Impact of Postprescription Review and Feedback. Open Forum Infectious Diseases. 2019;6(10).

15.	Gurung S, Kafle S, Dhungel B, Adhikari N, Shrestha UT, Adhikari B, et al. &Lt;p&gt;Detection of OXA-48 Gene in Carbapenem-Resistant &Lt;em&gt;Escherichia Coli&lt;/Em&gt; And &Lt;em&gt;Klebsiella Pneumoniae&lt;/Em&gt; From Urine Samples&lt;/P&gt. Infection and Drug Resistance. 2020;Volume 13:2311-21.

16.	Gallego M, Salazar‐Ospina L, Jiménez JN. The Modified Carbapenem Inactivation Method (mCIM): Highly Sensitive and Specific Low-Cost Tool to Assess Carbapenemase-Producing and Non-Producing in Gram-Negative Bacilli. Hechos Microbiológicos. 2022;13(2).

17.	Microorganisms Isolated from the Patients in the Intensive Care Unit and Their Antibiotic Susceptibilities. Annals of Clinical and Analytical Medicine. 2013;04(01).

18.	Aghamohammad S, Badmasti F, Shirazi AS, Dabiri H, Solgi H, Sabeti S, et al. Considerable rate of putative virulent phylo-groups in fecal carriage of extended-spectrum β-lactamase producing Escherichia coli. Infection, Genetics and Evolution. 2019;73:184-9.

19.	Aksaray SgS. Surveillance of antimicrobial resistance among Gram-negative isolates from intensive care units in eight hospitals in Turkey. Journal of Antimicrobial Chemotherapy. 2000;45(5):695-9.

20.	Alikhani A, Najafi N, Davoudi A, Tiroum S, Khademloo MR, Tayyebi ME, et al. Microbiological and Minimum Inhibitory Concentration Study of Ventilator-associated Pneumonia Agents in Two University-associated Hospital Intensive Care Units in Mazandaran. Archives of Clinical Infectious Diseases. 2012;8(1):8-13.

21.	Annamalai A, Gupta V, Jain S, Datta P. Increasing Resistance to Reserve Antibiotics: The Experience of a Tertiary Level Neonatal Intensive Care Unit. Journal of Tropical Pediatrics. 2020;67(1).

22.	Atray D, Sheethal S. Bacteriological Profile and Antibiotic Susceptibility Patterns of Gram-negative Bacilli Isolated from Lower Respiratory Tract Infections. Indian Journal of Medical Specialities. 2023;14(1):31-6.

23.	Azim NSA, Al-Harbi MA, Al-Zaban MI, Nofal MY, Somily AM. Prevalence and Antibiotic Susceptibility among Gram Negative Bacteria Isolated from Intensive Care Units at a Tertiary Care Hospital in Riyadh, Saudi Arabia. Journal of Pure and Applied Microbiology. 2019;13(1):201-8.

24.	Bandy A, Tantry B. ESBL Activity, MDR, and Carbapenem Resistance among Predominant Enterobacterales Isolated in 2019. Antibiotics. 2021;10(6):744.

25.	Bazaid AS, Aldarhami A, Gattan H, Barnawi H, Qanash H, Alsaif G, et al. Antibiogram of Urinary Tract Infections and Sepsis among Infants in Neonatal Intensive Care Unit. Children. 2022;9(5):629.

26.	Chang F, Wang X, Huang X, Liu X, Huang L. Analysis on Bacterial Distribution and Change of Drug Resistance Rate in ICUs Across Southwest China from 2018 to 2022. Infection and Drug Resistance. 2023;Volume 16:5685-96.

27.	Ding Y, Zhuang H, Zhou J, Xu L, Yang Y, He J, et al. Epidemiology and Genetic Characteristics of Carbapenem-Resistant Escherichia coli in Chinese Intensive Care Unit Analyzed by Whole-Genome Sequencing: a Prospective Observational Study. Microbiology Spectrum. 2023;11(2).

28.	Ergul AB, Isik H, Ay Altintop Y, Altuner Torun Y. A retrospective evaluation of blood cultures in a pediatric intensive care unit: a three year evaluation. Türk Pediatri Arşivi. 2017;52(3):154-61.

29.	Fahim NAE. Prevalence and antimicrobial susceptibility profile of multidrug-resistant bacteria among intensive care units patients at Ain Shams University Hospitals in Egypt—a retrospective study. Journal of the Egyptian Public Health Association. 2021;96(1).

30.	Friedland I, Stinson L, Ikaiddi M, Harm S, Woods GL. Resistance in Enterobacteriaceae: Results of a Multicenter Surveillance Study, 1995-2000. Infection Control &amp; Hospital Epidemiology. 2003;24(8):607-12.

31.	García-Cedrón D, De La Cruz Noriega M, Cabanillas-Chirinos L, Otiniano NM, Rojas-Villacorta W, Salvatierra-Espinola W, et al. Carbapenemase-Producing Bacteria Isolated from ICU Patients of a Peruvian Government Hospital during the COVID-19 Pandemic: A Descriptive Analysis. Medicina. 2023;59(10):1763.

32.	Gautam G, Satija S, Kaur R, Kumar A, Sharma D, Dhakad MS. Insight into the Burden of Antimicrobial Resistance among Bacterial Pathogens Isolated from Patients Admitted in ICUs of a Tertiary Care Hospital in India. Canadian Journal of Infectious Diseases and Medical Microbiology. 2024;2024:1-8.

33.	Ghenea AE, Zlatian OM, Cristea OM, Ungureanu A, Mititelu RR, Balasoiu AT, et al. TEM,CTX-M,SHV Genes in ESBL-Producing Escherichia coli and Klebsiella pneumoniae Isolated from Clinical Samples in a County Clinical Emergency Hospital Romania-Predominance of CTX-M-15. Antibiotics. 2022;11(4):503.

34.	Glupczynski Y, Delmée M, Goossens H, Struelens M. A Multicentre Survey of Antimicrobial Resistance in Gram-Negative Isolates from Belgian Intensive Care Units in 1994-1995. Acta Clinica Belgica. 1998;53(1):28-38.

35.	Golli A-L, Cristea OM, Zlatian O, Glodeanu A-D, Balasoiu AT, Ionescu M, et al. Prevalence of Multidrug-Resistant Pathogens Causing Bloodstream Infections in an Intensive Care Unit. Infection and Drug Resistance. 2022;Volume 15:5981-92.

36.	Gunseren F. A surveillance study of antimicrobial resistance of Gram-negative bacteria isolated from intensive care units in eight hospitals in Turkey. Journal of Antimicrobial Chemotherapy. 1999;43(3):373-8.

37.	Hamishehkar H, Shadmehr P, Mahmoodpoor A, Mashayekhi SO, Entezari-Maleki T. Antimicrobial susceptibility patterns among bacteria isolated from intensive care units of the largest teaching hospital at the northwest of Iran. Brazilian Journal of Pharmaceutical Sciences. 2016;52(3):403-12.

38.	Hanberger H. Antibiotic Susceptibility Among Aerobic Gram-negative Bacilli in Intensive Care Units in 5 European Countries. JAMA. 1999;281(1):67.

39.	İNcecİK Ş, SaltoĞLu N, Yaman A, Karayaylali İ, ÖZalevlİ M, GÜNdÜZ M, et al. The Problem of Antimicrobial Resistance in Nosocomial Medical and Surgical Intensive Care Units Infections in a University Hospital; a Two-Year Prospective Study. Turkish Journal of Medical Sciences. 2009.

40.	Jean S-S, Hsueh P-R, Lee W-S, Yu K-W, Liao C-H, Chang F-Y, et al. Carbapenem susceptibilities and non-susceptibility concordance to different carbapenems amongst clinically important Gram-negative bacteria isolated from intensive care units in Taiwan: Results from the Surveillance of Multicentre Antimicrobial Resistance in Taiwan (SMART) in 2009. International Journal of Antimicrobial Agents. 2013;41(5):457-62.

41.	Jean S-S, Lee W-S, Bai K-J, Yu K-W, Hsu C-W, Yu K-W, et al. Carbapenem susceptibility among Escherichia coli, Klebsiella pneumoniae, and Enterobacter cloacae isolates obtained from patients in intensive care units in Taiwan in 2005, 2007, and 2009. Diagnostic Microbiology and Infectious Disease. 2015;81(4):290-5.

42.	Jean S-S, Lee W-S, Yu K-W, Liao C-H, Hsu C-W, Chang F-Y, et al. Rates of susceptibility of carbapenems, ceftobiprole, and colistin against clinically important bacteria collected from intensive care units in 2007: Results from the Surveillance of Multicenter Antimicrobial Resistance in Taiwan (SMART). Journal of Microbiology, Immunology and Infection. 2016;49(6):969-76.

43.	Khare V. Study on MICs of Tigecycline in Clinical Isolates of Carbapenem Resistant Enterobacteriaceae (CRE) at a Tertiary Care Centre in North India. JOURNAL OF CLINICAL AND DIAGNOSTIC RESEARCH. 2017.

44.	Lai C-C, Chen Y-S, Lee N-Y, Tang H-J, Lee SS-J, Lin C-F, et al. &lt;p&gt;Susceptibility rates of clinically important bacteria collected from intensive care units against colistin, carbapenems, and other comparative agents: results from the Surveillance of Multicenter Antimicrobial Resistance in Taiwan (SMART)&lt;/p&gt. Infection and Drug Resistance. 2019;Volume 12:627-40.

45.	Liu Q, Liu L, Li Y, Chen X, Yan Q, Liu W-e. &lt;p&gt;Fecal Carriage and Epidemiology of Carbapenem-Resistant &lt;em&gt;Enterobacteriaceae&lt;/em&gt; Among Hospitalized Patients in a University Hospital&lt;/p&gt. Infection and Drug Resistance. 2019;Volume 12:3935-42.

46.	Mohammadtaheri Z, Pourpaki M, Mohammadi F, Namdar R, Masjedi M-R. Surveillance of Antimicrobial Susceptibility among Bacterial Isolates from Intensive Care Unit Patients of a Tertiary-Care University Hospital in Iran: 2006–2009. Chemotherapy. 2010;56(6):478-84.

47.	Mw M. Antibiogram of Catheter Associated Bacterial Pathogens in Urinary Tract Infection among Pediatrics Patients in Pakistan. International Journal of Pharmacognosy &amp; Chinese Medicine. 2023;7(2):1-11.

48.	Nusrat T, Akter N, Haque M, Rahman NAA, Dewanjee AK, Ahmed S, et al. Comparative Study of CDST &amp; Multiplex PCR to Detect MBL Producing Gram-Negative Bacilli among VAP Patients Admitted in a Public Medical College Hospital of Bangladesh. Pathogens. 2019;8(3):151.

49.	Saseedharan S. Act Fast as Time Is Less: High Faecal Carriage of CarbapenemResistant Enterobacteriaceae in Critical Care Patients. JOURNAL OF CLINICAL AND DIAGNOSTIC RESEARCH. 2016.

50.	Singh N. Prevalence of ESBL in Escherichia coli Isolates Among ICU Patients in a Tertiary Care Hospital. JOURNAL OF CLINICAL AND DIAGNOSTIC RESEARCH. 2016.

51.	Tan R, Liu J, Li M, Huang J, Sun J, Qu H. Epidemiology and antimicrobial resistance among commonly encountered bacteria associated with infections and colonization in intensive care units in a university-affiliated hospital in Shanghai. Journal of Microbiology, Immunology and Infection. 2014;47(2):87-94.

52.	Xiao R, Li Y, Liu X, Ding Y, Lai J, Li Y, et al. Antibiotic susceptibility of Escherichia coli isolated from neonates admitted to neonatal intensive care units across China from 2015 to 2020. Frontiers in Cellular and Infection Microbiology. 2023;13.

53.	Xie J, Peters BM, Li B, Li L, Yu G, Xu Z, et al. Clinical features and antimicrobial resistance profiles of important Enterobacteriaceae pathogens in Guangzhou representative of Southern China, 2001–2015. Microbial Pathogenesis. 2017;107:206-11.

54.	Yang Q. Antimicrobial susceptibility profiles of Gram-negative bacilli isolated from patients with hepatobiliary infections in China: results from the study for monitoring antimicrobial resistance trends (SMART), 2006–2013. Morressier; 2016.

55.	Yang Q, Zhang H, Yu Y, Kong H, Duan Q, Wang Y, et al. In Vitro Activity of Imipenem/Relebactam Against Enterobacteriaceae Isolates Obtained from Intra-abdominal, Respiratory Tract, and Urinary Tract Infections in China: Study for Monitoring Antimicrobial Resistance Trends (SMART), 2015–2018. Clinical Infectious Diseases. 2020;71(Supplement_4):S427-S35.

56.	Yin K, Liu L, Fan G. Classification and Drug Resistance Analysis of Pathogenic Bacteria in Patients with Bacterial Pneumonia in Emergency Intensive Care Unit. Contrast Media &amp; Molecular Imaging. 2022;2022(1).

57.	Yücesoy M, yulu N, Kocagöz S, Ünal S, Çetin S, Çalangu S. Antimicrobial Resistance of Gram-Negative Isolates from Intensive Care Units in Turkey: Comparison to Previous Three Years. Journal of Chemotherapy. 2000;12(4):294-8.
